# Cerebrospinal beta-amyloid peptides(1-40) and (1-42) in severe preeclampsia and HELLP syndrome – a pilot study

**DOI:** 10.1038/s41598-020-62805-2

**Published:** 2020-04-01

**Authors:** Wolfgang Lederer, Helene Schaffenrath, Cristina Alomar-Dominguez, Julia Thaler, Raffaella Fantin, Lucie Dostal, Guenther Putz, Christian Humpel

**Affiliations:** 1 Medical University of Innsbruck, Department of Anesthesiology and Critical Care Medicine, Innsbruck, 6020 Austria; 2 Medical University of Innsbruck, Department of Gynecology and Obstetrics, Innsbruck, 6020 Austria; 3 Medical University of Innsbruck, Department of Medical Statistics, Informatics and Health Economics, Innsbruck, 6020 Austria; 4 Medical University of Innsbruck Department of Psychiatry, Psychotherapy and Psychosomatics, Innsbruck, 6020 Austria

**Keywords:** Biomarkers, Health care

## Abstract

During pregnancy, substantial alterations in cerebral plasticity, vascular remodeling and neuronal growth occur in the maternal brain. We investigated whether concentrations of selected neurodiagnostic biomarkers in the cerebrospinal fluid of women with preeclampsia/HELLP syndrome differ from those in healthy controls using enzyme-linked immunosorbent assay technique. We found that **t**au protein concentrations (p = 0.016) and phospho-tau/tau ratio (p < 0.001) in cerebrospinal fluid were significantly lower in 39 preeclamptic women compared to 44 healthy controls during third trimester of pregnancy. Beta-amyloid(1-40)/(1-42) ratio was significantly higher in HELLP syndrome than in severe preeclampsia (8.49 + 2.73 vs. 4.71 + 1.65; p = 0.007). We conclude that beta-amyloid(1-40)/(1-42) ratio in cerebrospinal fluid can discriminate severe preeclampsia and HELLP syndrome. High beta-amyloid peptide and low **t**au protein concentrations are associated with impaired development of the materno-feto-placental unit and correlate with placental dysfunction.

## Introduction

During pregnancy, the invasion of trophoblast cells into maternal tissue of the uterus and the conversion of spiral arteries into wide sinusoids with low resistance and high flow are paramount for normal placental development^1^. In preeclampsia (PE) placental development is impaired by defective deep placentation, platelet and thrombin activation, intravascular inflammation, endothelial dysfunction and imbalanced angiogenesis^2^. Altered expression of proteins comes along with excess of antiangiogenic substances such as soluble fms-like tyrosine kinase-1 (sFlt-1) and soluble endoglin (sEng) and decreased levels of proangiogenic substances like placental growth factor (PlGF) and vascular endothelial growth factor A (VEGF-A)^3^. Ciampa *et al*. observed in 13 patients with PE altered concentrations of proteins related to signaling pathways important for vascular remodeling, inflammation, and neuronal growth^4^.

Recent studies have shown that PE shares pathophysiologic features with recognized misfolding disorders and aggregation of proteins^4–8^. There are several dysregulated proteins in PE but it is not clear whether aggregated proteins induce defective trophoblast invasion^4^. D’Souza *et al*. reported that neurotrophic factors influence the development of the materno-feto-placental unit during pregnancy^9^. Altered blood-brain barrier and impaired cerebral autoregulation may affect erebral blood flow in the maternal brain^10^. Aggregated beta-amyloid peptides were observed in PE as well as in Alzheimer’s disease^7^. The presence of beta-amyloid aggregates in placentas of women with PE and intrauterine growth restriction (IUGR) further supports the notion that this condition goes with protein conformational disorders^6,11^. Moreover, it was observed that short peptides occupying the self-recognition sites of beta-amyloid inhibit beta-amyloid aggregation^8^.

The aim of this study was to determine whether CSF concentrations of beta-amyloid peptides and tau protein differ between women with PE and women with HELLP syndrome as compared to healthy pregnant women during the third trimester of pregnancy.

## Results

### Patient characteristics

**Figure 1:**
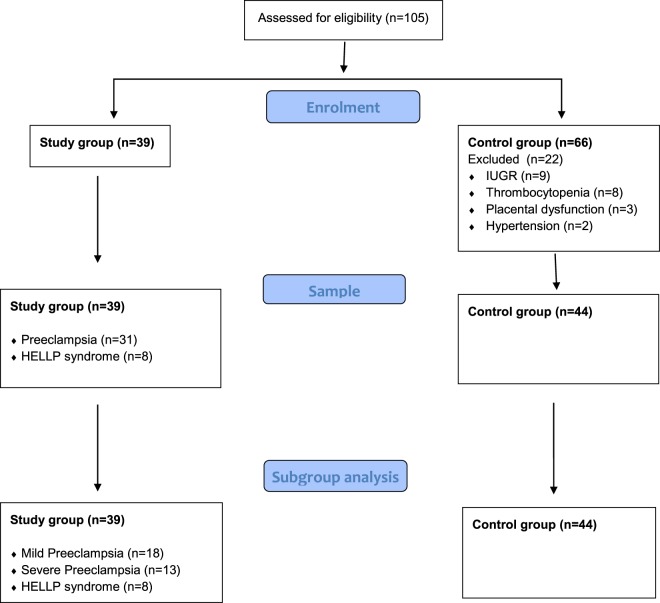
CONSORT 2010 Flow Diagram of patient enrolment.

A total of 105 pregnant women who underwent spinal anesthesia participated; 39 women with PE/HELLP were consecutively assigned to a prospective research cohort. Forty-four of 66 pregnant women served as controls (Fig. 1). Demographic data including age, height, current weight and BMI were comparable between study group and controls. The number of previous pregnancies, miscarriages and parities did not differ between the two groups, but weeks of gestation were significantly fewer in women with PE/HELLP syndrome than in controls (33.4 + 3.4 vs. 38.1 + 1.1; p < 0.001).

### Cardiorespiratory parameters and blood chemistry

In the study group we distinguished mild PE (n = 18), severe PE (n = 13) and HELLP syndrome (n = 8). According to the underlying pathomechanism systolic blood pressure (167.1 + 16.9 vs. 121.4 + 8.4; p < 0.001), diastolic blood pressure (103.9 + 20.4 vs. 77.8 + 8.4; p < 0.001) and pulse rate (82.5 + 17.0 vs. 89.8 + 13.4; p = 0.023) differed significantly between PE and HELLP syndrome and controls. In HELLP syndrome platelet count (85.6 × 10^9^/L + 25.5) was significantly lower than for mild PE (171.3 × 10^9^/L + 54.8; p = 0.002) or severe PE (178.0 × 10^9^/L + 47.1; p = 0.001). In addition, sGOT in HELLP syndrome (263.9 U/L + 470.4) was significantly higher than in mild PE (29.3 U/L + 16.4; P < 0.001) or severe PE (33.5 U/L + 15.0; p = 0.005).

### Health status of the newly born

Mean baby weight differed significantly between mild PE (2,362 g + 546.9) and severe PE (1,095 g + 359.2; p < 0.001), as well as between severe PE and HELLP syndrome (1,993 g + 543.7; p = 0.018). In a post hoc subgroup analysis placental weight (p = 0.021) and baby weight (p = 0.001) were significantly lower in cases with beta-amyloid(1-40) exceeding 5000 pg/mL compared to beta amyloid(1-40) beyond 5000 pg/mL.

### Biomarkers

CSF concentrations of tau protein were significantly lower in 39 women with PE and HELLP syndrome than in controls (p = 0.016). Phospho-tau-181/tau ratio was significantly higher in women with PE/HELLP (p < 0.001). In the study group median concentrations of beta-amyloid peptides(1-40) were higher in HELLP syndrome (9,683 pg/mL ± 5,643; p = 0.023) than for severe PE (4,836 pg/mL ± 2,004), and the beta-amyloid(1-40)/(1-42) ratio differed significantly (8.49  ±  2.73 vs. 4.71  ±  1.65; p = 0.007) (Table 1).

**Table 1:** CSF beta-amyloid peptides (Aß(1-42) and Aß(1-40)), tau and phospho-tau-181 (p-tau-181) protein levels in 39 women in the study group with either mild preeclampsia (PE) or severe PE or HELLP syndrome, compared to 44 healthy controls at delivery.

	Mild PE	Severe PE	HELLP syndrome	controls
	(n = 18)	(n = 13)	(n = 8)	(n = 44)
***CSF biomarkers***				
Aβ(1-42) [pg/ml]	1,158 ± 444.7	1,041 ± 143.3	1,071 ± 391.8	1,090 ± 166.3
Aβ(1-40) [pg/ml]	7,691 ± 3,837	4,836 ± 2,004	9,683 ± 5,463	6,329 ± 2,114
Aβ(1-40)/(1-42) ratio	6.40 ± 1.80	4.71 ± 1.65	8.49 ± 2.73	5.81 ± 1.97
Tau [pg/ml]	246.7 ± 136.0	271.8 ± 127.8	240.1 ± 109.7	312.9 ± 127.9
P-tau-181 [pg/ml]	42.9 ± 18.8	47.7 ± 19.1	44.9 ± 18.1	50.5 ± 18.1
P-tau/tau ratio	0.19 ± 0.03	0.18 ± 0.02	0.19 ± 0.01	0.16 ± 0.01
***Serum biomarkers***				
sFlt-1 [pg/ml]	13,819 ± 7690	14,369 ± 10,795	10,853 ± 7,882	3,554 ± 2,185
PlGF [pg/ml]	135.2 ± 143.0	105.2 ± 156.2	350.8 ± 767.5	619.7 ± 398.9
sFlt-1/PlGF ratio	102.2 ± 53.4	136.6 ± 69.1	30.9 ± 10.3	5.7 ± 5.5

Values are expressed as median and standard deviation.

Beta-amyloid(1-40) correlated with serum markers sFlt-1 (r_s_ = −0.536; p = 0.001) and PlGF (r_s_ = 0.357; p = 0.042). Tau protein correlated with serum markers sFlt-1 (r_s_ = −0.330; p = 0.040) and PlGF (r_s_ = 0.356; p = 0.028). Beta-amyloid(1-40)/(1-42) ratio was significantly higher in HELLP syndrome than in severe PE (8.49 + 2.73 vs. 4.71 + 1.65; p = 0.007) (Table 1). Using post-hoc analysis with the Dunn-Bonferroni test, the beta-amyloid(1-40)/(1-42) ratio differed significantly between HELLP syndrome and severe PE (p = 0.007) and between HELLP syndrome and controls (p = 0.049) (Table 2).

**Table 2:** CSF beta-amyloid peptide(1-42)/(1-40) ratio among 39 women in the study group with either mild preeclampsia (PE) or severe PE or HELLP syndrome and 44 healthy controls at delivery.

	Mild PE	Severe PE	HELLP syndrome	Controls
	(n = 18)	(n = 13)	(n = 8)	(n = 44)
Mild PE		0.128	0.735	1.000
Severe PE			**0.006**	0.805
HELLP syndrome				**0.049**

Statistical comparison was made using the Dunn-Bonferroni test. Significant p values are indicated in bold.

## Discussion

In our study mean concentrations of beta-amyloid peptides(1-40) were higher in women with HELLP syndrome than in preeclamptic women and healthy controls. Human placenta and thrombocytes abundantly express amyloid precursor protein (APP)^5,6^. Presumably, thrombocytopenia influences metabolism of beta-amyloid(1-42) and (1-40)^12^. Although the histopathologic profile and the range of placental lesions differ between PE and HELLP syndrome^13^, it remains a matter of ongoing debate whether HELLP syndrome is a severe form of PE or a separate disease. We found that beta-amyloid(1-40)/(1-42) ratio can discriminate between severe PE and HELLP syndrome. The findings of our study support the hypothesis that HELLP syndrome is a distinct disease pattern and not simply a variety of severe PE.

In our study, median CSF concentrations of tau protein and the phospho-tau/tau ratio were significantly lower in women with PE and HELLP syndrome than in healthy controls. This is in agreement with recently reported diminished CSF tau and phospho-tau-181 protein concentrations in patients with placental dysfunction^14,15^. During normal pregnancy there is an increase in tau protein concentrations^16^. Presumably, diminished CSF concentrations in PE and HELLP syndrome imply fewer maternal brain adaptations. Virtanen *et al*. observed an inhibitory effect on tubule formation in third trimester preeclamptic women^17^. Serum concentrations of angiogenic factors sFlt-1 levels were higher and PlGF were lower in third trimester preeclamptic women compared to healthy controls with increasing sFlt-1/PlGF ratio between the first and the third trimester^18^. Unfortunately, sFlt-1 and PlGF have limited sensitivity for stratification of women with suspected PE^19^. PE still lacks a reliable, early means of diagnosis or prediction, and a safe and effective therapy^20^, So far, the diagnosis of PE and HELLP syndrome is mostly based on clinical findings and increasing sFlt-1/PlGF ratio. In particular, the onset threshold of plasma levels of the sFlt-1/PlGF ratio proved to be a valuable screening tool for detecting the imminent onset of PE within four weeks after blood sampling during the second trimester, namely at 19 to 31 weeks^21^. Potentially, detection of amyloid-targeting fluorophores in the urine may contribute to early identification of PE patients during pregnancy^22^. Affintiy for conformational antibodies raised against aggregated beta-amyloid peptides and dysregulation in the APP proteolytic pathway may offer future diagnostic and therapeutic options^6,8^. Neuron-specific enolase and S100B were reported to be increased before clinical development of PE can be verified^23^. This supports the hypothesis that altered neural remodeling in the maternal brain precedes impaired development of the placenta.

Pregnancy renders substantial changes in maternal brain, primarily reduction in gray matter volume in regions subserving social cognition^24^. Decrease in brain size begins after placental implantation^25^. It reaches maximum at term and it endures for at least 2 years post-partum^25,26^. Structural and functional changes accompany fundamental behavioural adaptations, stimulating to progress from self-involved individuality to carying motherhood^26^. Changes in the maternal brain during pregnancy cannot be explained by endocrine and environmental factors alone^27^. Van Dijk *et al*. reported that the PE-susceptibility gene STOX1 controls a conserved pathway shared between placenta and brain^28^. STOX1A correlated with severity of disease and was associated with increased amyloid-beta protein precursor processing in neural cells and trophoblast cells^28^. Vaiman and Miralles hypothesized that selective inhibition of one of the two isoforms STOX1A and STOX1B could possibly improve outcome in severe PE^29^.

In our study angiogenic factors in serum correlate with CSF beta-amyloid(1-40) and tau protein. Our findings question the current perspective on etiology of PE and of HELLP syndrome and support the hypothesis that neurotrophic factors influence the development of the materno-feto-placental unit during pregnancy. However, there are some limitations. First and foremost, the number of enrolled women with PE and HELLP syndrome is small as data were only available on women who have already developed clinically evident disease and who underwent surgical or obstetrical operations in spinal anesthesia. We are aware that routine lumbar puncture for diagnosis of PE and HELLP syndrome is not practical during pregnancy. Furthermore, treatment is limited mostly to maternal stabilization and timely delivery by medically induced labor or cesarean section^2^. In women with PE and HELLP syndrome delivery by cesarean section was on average four weeks of gestation earlier than in controls, thus partly explaining the low values for placental weight, baby weight and Apgar score. Duration of storage of samples varied among women enrolled and might have biased measurement results. Sampling of patient CSF was performed mostly during the daytime. Observer bias among pediatricians and midwives might have influenced assessment of Apgar Scores and placental weight. Furthermore, rating of perceived stress and physical activity depended on subjective justification of participating women and might have been influenced by current events. Larger trials focusing on placental morphology and function are needed to draw definitive conclusions.

In conclusion we observed that beta-amyloid(1-40)/(1-42) ratio in cerebrospinal fluid can discriminate severe PE and HELLP syndrome. High beta-amyloid peptide and low tau protein concentrations are associated with impaired development of the materno-feto-placental unit and correlate with placental dysfunction.

## Materials and methods

Women with PE/HELLP syndrome during pregnancy who underwent spinal puncture for regional anesthesia were consecutively enroled during normal work time in a prospective cross-sectional research at the Department of Gynecology, Innsbruck Medical University Hospital. Healthy women with uncomplicated pregnancies who underwent a cesarean section under spinal block served as controls.

### Ethical approval & patients consent

The Ethics Committee of Innsbruck Medical University approved this study (AN2017-0073 371/4.25). Written informed consent was obtained from all patients before participation with the understanding that anonymized data would be published in a scientific journal. All methods were performed in accordance with the relevant guidelines and regulations.

### Study design and study population

Anesthetists from obstetrical anesthesia screened patients by medical history before participation. Blood chemistry (blood sugar, hemoglobin, serum protein) and kidney function parameters (blood urea nitrogen, creatinine, glomerular filtration rate, and urinary protein) were obtained from medical records. Circulatory and respiratory status (systolic blood pressure, pulse rate and oxygen saturation) were recorded before and during anesthesia. Perceived stress and physical activity were inquired, as they may affect incidence and severity of PE^30^. We used a 5-point Likert scale with (1, very much; 2, much; 3, moderate; 4, poor; 5, very poor) for individual grading of conceptualization of estimated stress. Demographics, clinical characteristics and laboratory findings for participating women were recorded on a working chart.

Inclusion criteria for the study group were: pregnant women, 18 years of age or older, with evidence of PE/HELLP syndrome verified by clinical signs of hypertension, proteinuria and edema and, if available, positive sFlt-1/PlGF ratio^31^. Exclusion criteria for the study group were: lacking written consent. Inclusion criteria for the control group were: pregnant women, 18 years of age or older, in good general health. Participants were on no chronic medication other than multivitamins, magnesium and iron supplementation. Subjects were in particular free from past or present major psychiatric disorders other than mild depression, major neurological disease, gestational diabetes, and chronic renal and hepatic disease. Exclusion criteria for the control group were: women suffering from hypertension in pregnancy, thrombocytopenia, proteinuria, evidence of placental dysfunction, IUGR, as well as women with a history of PE or HELLP syndrome, cases lacking written consent. All study findings and documents were handled in strictest confidence. Enrolment of patients, data collection and analysis followed the CONSORT 2010 checklist of information to include when reporting a randomized trial^32^. Recruitment of patients depended on pre-determined sample sizes (quota sampling according to power analysis) stratified by clinical diagnosis and voluntary participation.

### Sample collection

All pregnant women undergoing a spinal block with direct access to CSF and meeting the inclusion criteria were eligible as study subjects. Spinal anesthesia was performed according to standard operating procedures of the Department of Anesthesiology and Critical Care Medicine^33^. Following skin disinfection, a 20-gauge introducer needle (B Braun, 34209 Melsungen, Germany) was inserted into the mid-line lumbar region and directed towards the interspinous ligament. Then, a 25-gauge needle (Spinocan^®^ pencil-point spinal needle, B Braun, 34209 Melsungen, Germany) was inserted through the introducer into the subarachnoid space and the trocar was removed. Before administration of the intrathecal local anesthetic one milliliter CSF was collected in a sterile polypropylene tube^34^. Immediately after transport to the psychiatric laboratory, CSF samples were frozen and stored at minus 80 °C until the assays were performed.

### Definitions

- Body mass index (BMI), calculated by dividing weight in kilograms by the square of height in meters.
- Physical activity, defined as (>30 minutes, very much; up to 30 minutes, much; 20 minutes, moderate; 10 minutes, poor; and <10 minutes, very poor) considering the World Health Organisation recommendations for daily physical activity for adults aged 18–64 years^35^.
- Placental dysfunction, abnormal uteroplacental and fetoplacental circulation assessed by Doppler sonography using Grannum classification graded as 0 (homogeneous), I (subtle), II (marked), III (confluent)^36^.
- HELLP syndrome, characterized by haemolysis, elevated liver enzymes and low platelets^37^.
- PE, systemic syndrome with hypertension exceeding 140/90 mmHg presenting beyond 20 weeks of gestation and proteinuria >300 mg protein in a 24 h urine collection or 1 + (0.3 g/L) on urine dipstick^37^.
- Early-onset PE, hypertension, proteinuria, placental dysfunction due to poor placental perfusion and reduction of placental volume, IUGR and low birth weight occurring before 34 weeks of gestation^37^.
- Mild PE, proteinuria and systolic blood pressure between 140 and 160 mmHg or diastolic blood pressure between 90 and 110 mmHg.
- Severe PE, proteinuria and blood pressure exceeding 160 mmHg systolic or 110 mmHg diastolic, frequently combined with elevated liver enzymes^37^.
- sFlt-1/PlGF ratio, calculated dividing antiangiogenic factor sFlt-1 by proangiogenic factor PlGF^33^.
- Thrombocytopenia in pregnancy, defined as a platelet count <100 × 10^9^/L^38^.

### Processing and analytical techniques

CSF biochemical markers were detected in cell-free samples using enzyme-linked immunosorbent assay (ELISA) assays (Fujrebio, formerly Innogenetics) that can reliably detect even low levels of CSF biomarkers based on several well-defined monoclonal tau antibodies for normal and abnormal forms of CSF tau protein. For analysis, all CSF samples were thawed and concentrations of beta-amyloid(1-42) and (1-40), total tau and phospho-tau-181 proteins (Assay Innotest^®^, Fujirebio Europe, B-9052 Ghent, Belgium) measured by ELISA according to the manufacturer’s instructions.

### Statistical analysis

Primary study endpoint was to evaluate selected CSF neurodiagnostic biomarkers in women with PE/HELLP syndrome. Secondary study endpoint was to detect differences in CSF biomarkers between preeclamptic women and women with HELLP syndrome. Our H0 hypothesis was: there are no differences in CSF concentrations of beta-amyloid(1-42) or (1-40), total tau or phospho-tau-181 proteins between women with PE and HELLP syndrome and normal pregnancies. In order to compensate for individual variations in beta-amyloid production the beta-amyloid(1-42)/(1-40) ratio was calculated.

The sample size was calculated for an alpha error of 0.05 and a power of 80% (beta error of 0.2) to detect significant differences between CSF biomarkers of women with PE/HELLP syndrome and women with normal pregnancy using nQueryAdvisor v.7.0 software. Calculations were conducted with SPSS 25 (IBM SPSS Statistics Standard) using the T test or the Mann-Whitney U test, as indicated. For multiple pairwise comparisons between more than two subgroups, the Kruskal-Wallis test was applied with Bonferroni correction. A *post hoc* subgroup analysis was performed for beta-amyloid(1-40) <5000 pg/mL and for beta amyloid(1-40) >5000 pg/mL. Group comparisons of the beta-amyloid(1-42)/(1-40) ratio were assessed by analysis of variance followed by the Dunn-Bonferroni test^39^. Pearson’s correlations (two-tailed, bivariate) were calculated for CSF marker levels and blood sugar, proteinuria, systolic blood pressure and body weight as expressed in BMI. Correlations with either stress or physical exercise values were established using the Spearman’s rank correlation coefficient. Statistical significance was deemed when p < 0.05.

## Supplementary information

Supplementary Dataset 1.

## Data Availability

Supplementary Information in an Additional File 1 is available in persistent web link to datasets. Further data and materials are available from the corresponding author on reasonable request.
