# Supplementary material for: Cerebrospinal beta-amyloid peptides(1-40) and (1-42) in severe preeclampsia and HELLP syndrome – a pilot study
Source: Sci Rep. 2020 Apr 1;10:5783. doi: 10.1038/s41598-020-62805-2 (PMC7113242; doi:10.1038/s41598-020-62805-2)

Cerebrospinal beta-amyloid peptides(1-40) and (1-42) in severe preeclampsia and HELLP syndrome – pilot study

Wolfgang Lederer[1,+,*], Helene Schaffenrath[2], Cristina Alomar-Dominguez[1,+], Julia Thaler[1], Raffaella Fantin[1], Lucie Dostal[3], Guenther Putz[1], Christian Humpel[4,+]

[1] Department of Anesthesiology and Critical Care Medicine, Medical University of Innsbruck

[2] Department of Gynecology and Obstetrics, Medical University of Innsbruck

[3] Department of Medical Statistics, Informatics and Health Economics, Medical University of Innsbruck

[4] Department of Psychiatry, Psychotherapy and Psychosomatics, Medical University of Innsbruck

Graph

Graph - Scatter of ßA1_40 APP - September 25, 2019

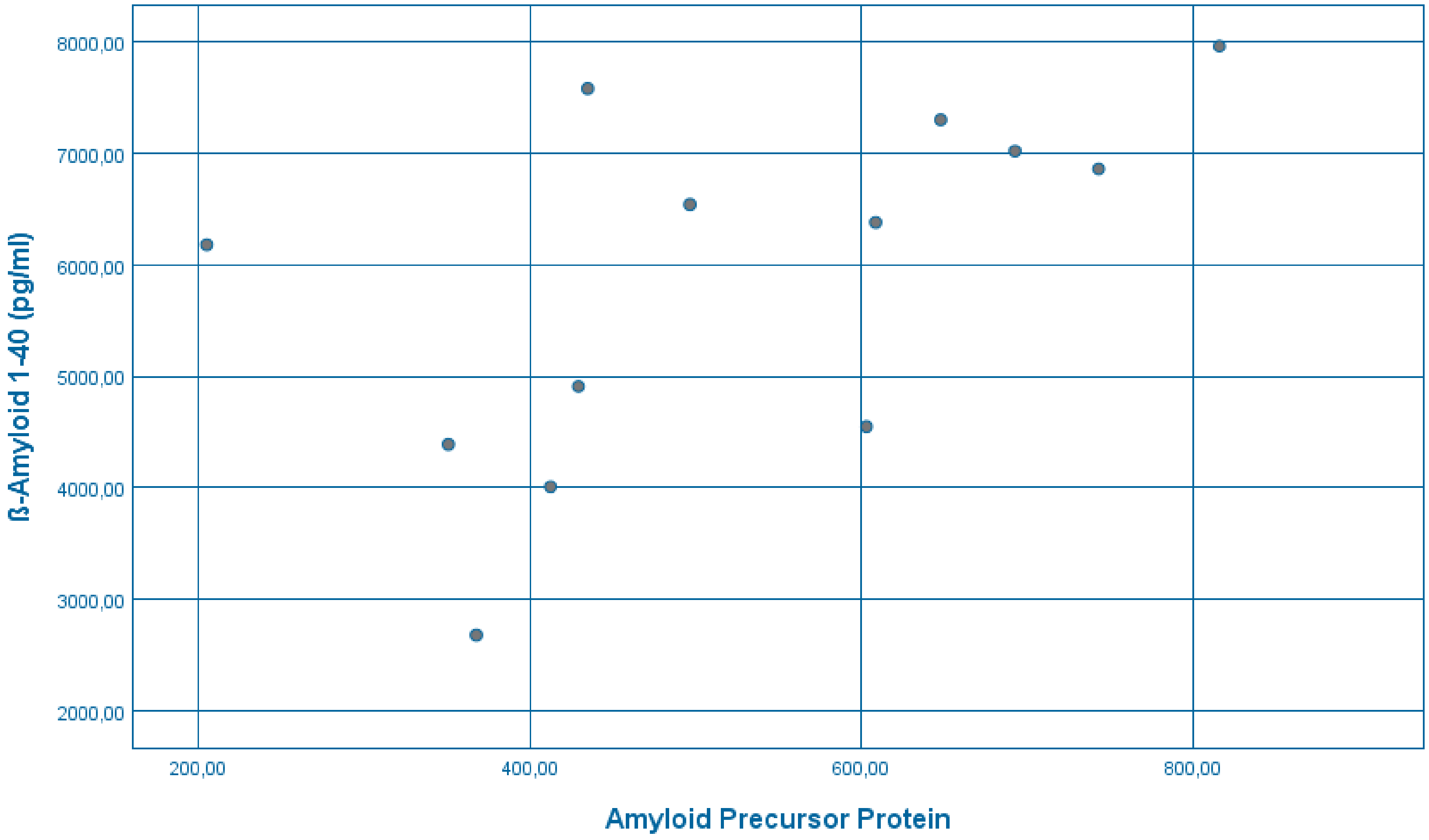

Graph

Graph - Scatter of ßA1_40 ßA1_42 - September 25, 2019

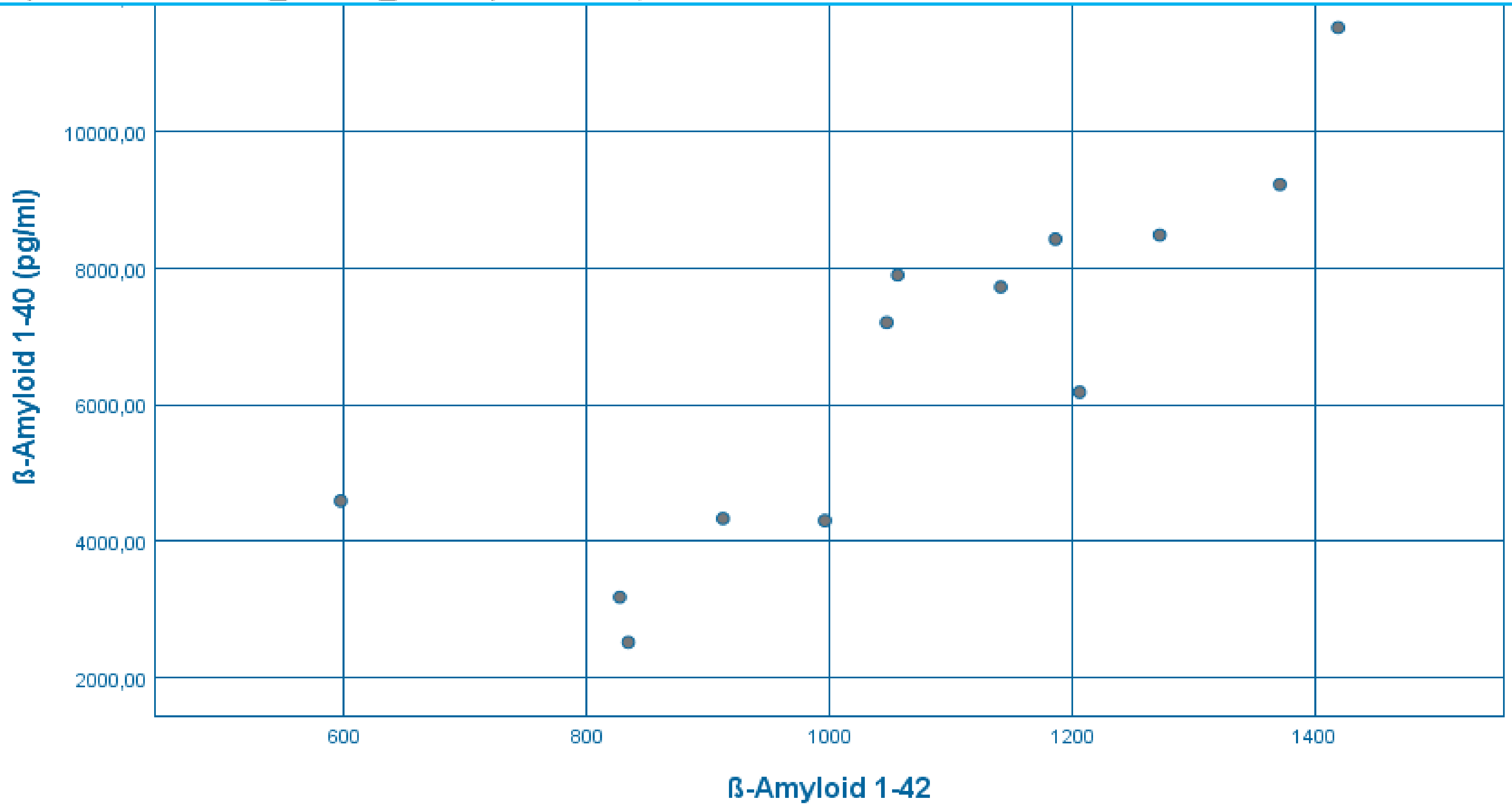

Graph

Graph - Scatter of Tau ßA1_42 - September 25, 2019

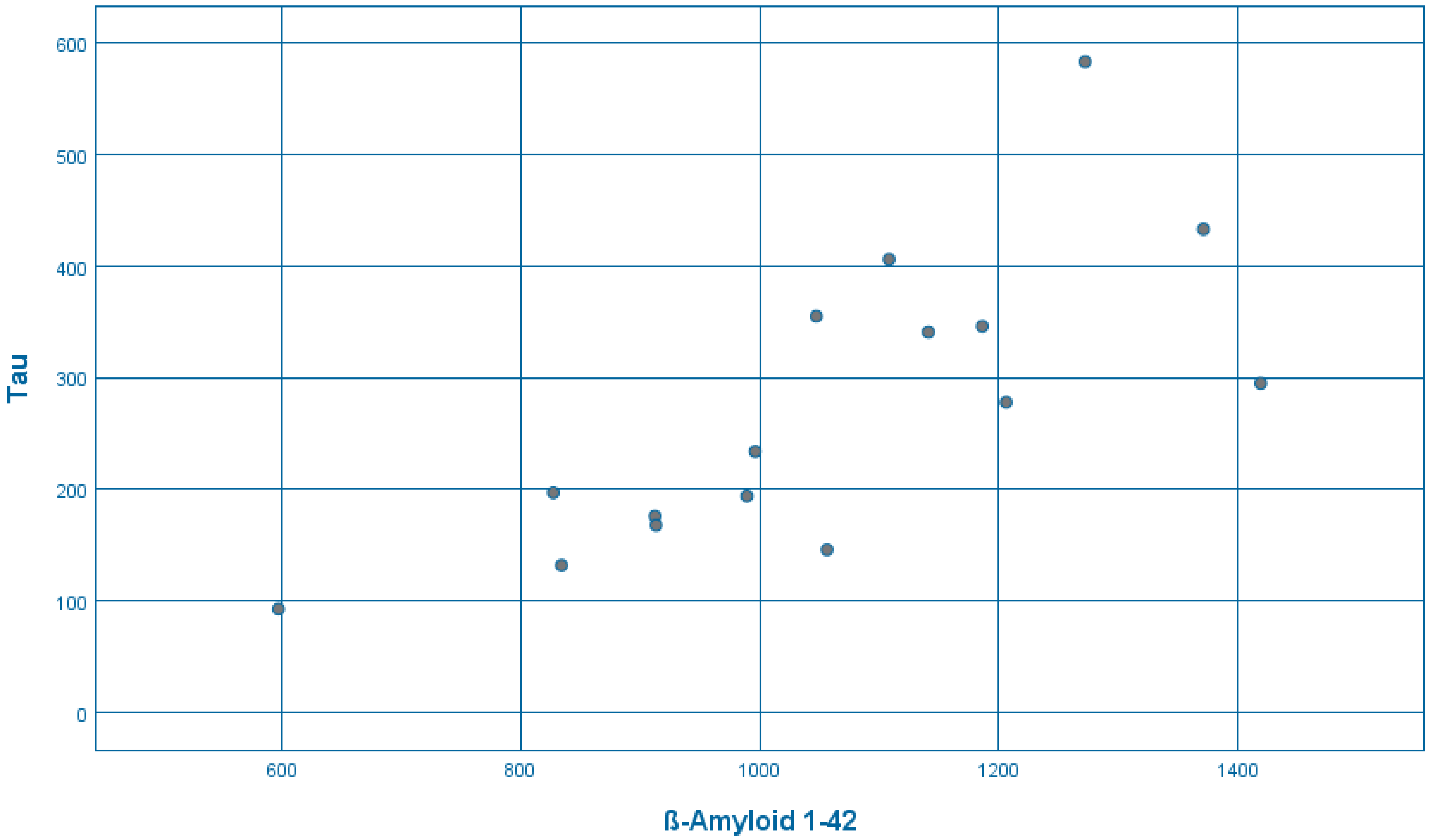

Graph

Graph - Scatter of pTau181 ßA1_42 - September 25, 2019

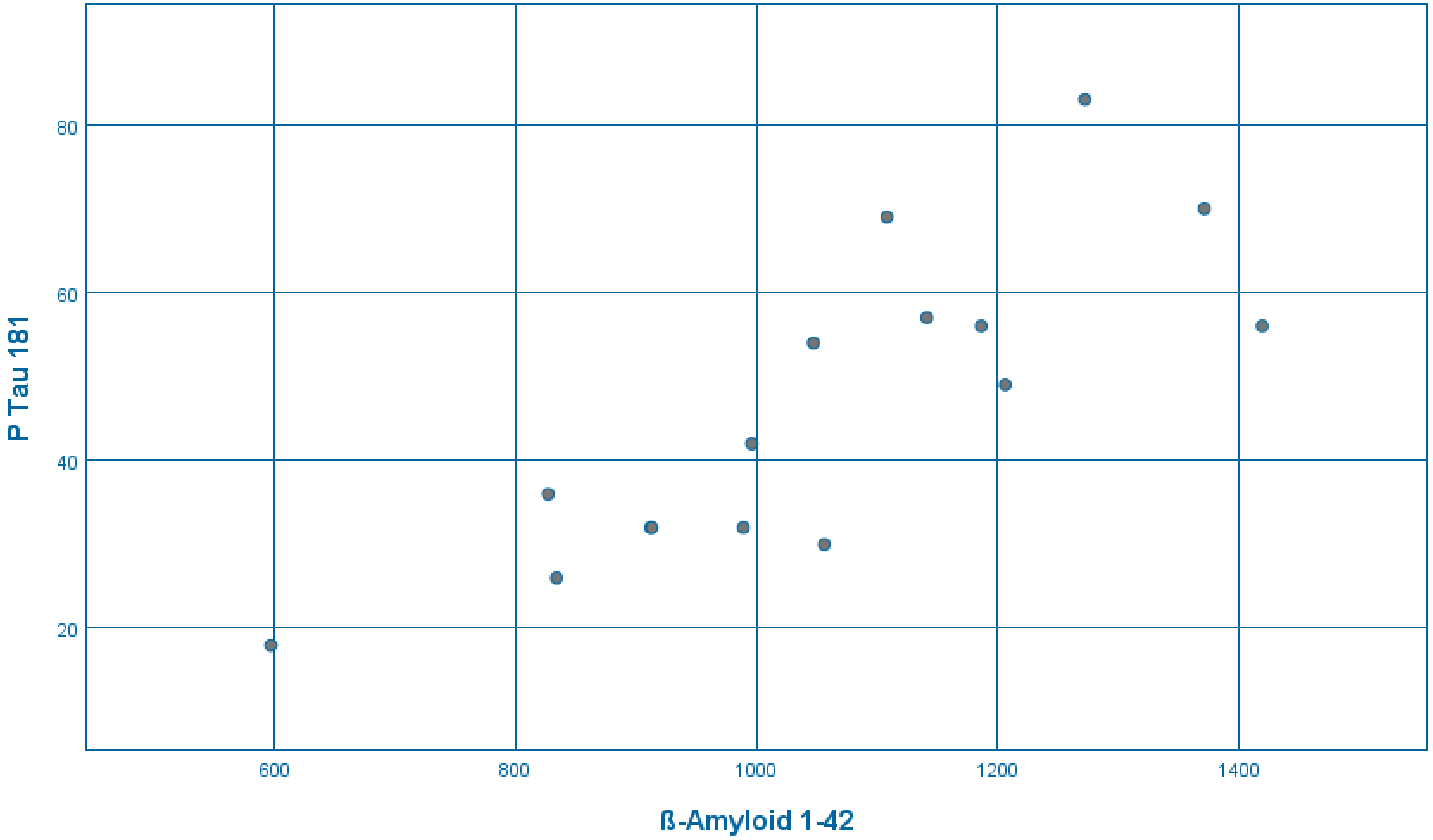

Graph

Graph - Scatter of Tau ßA1_40 - September 25, 2019

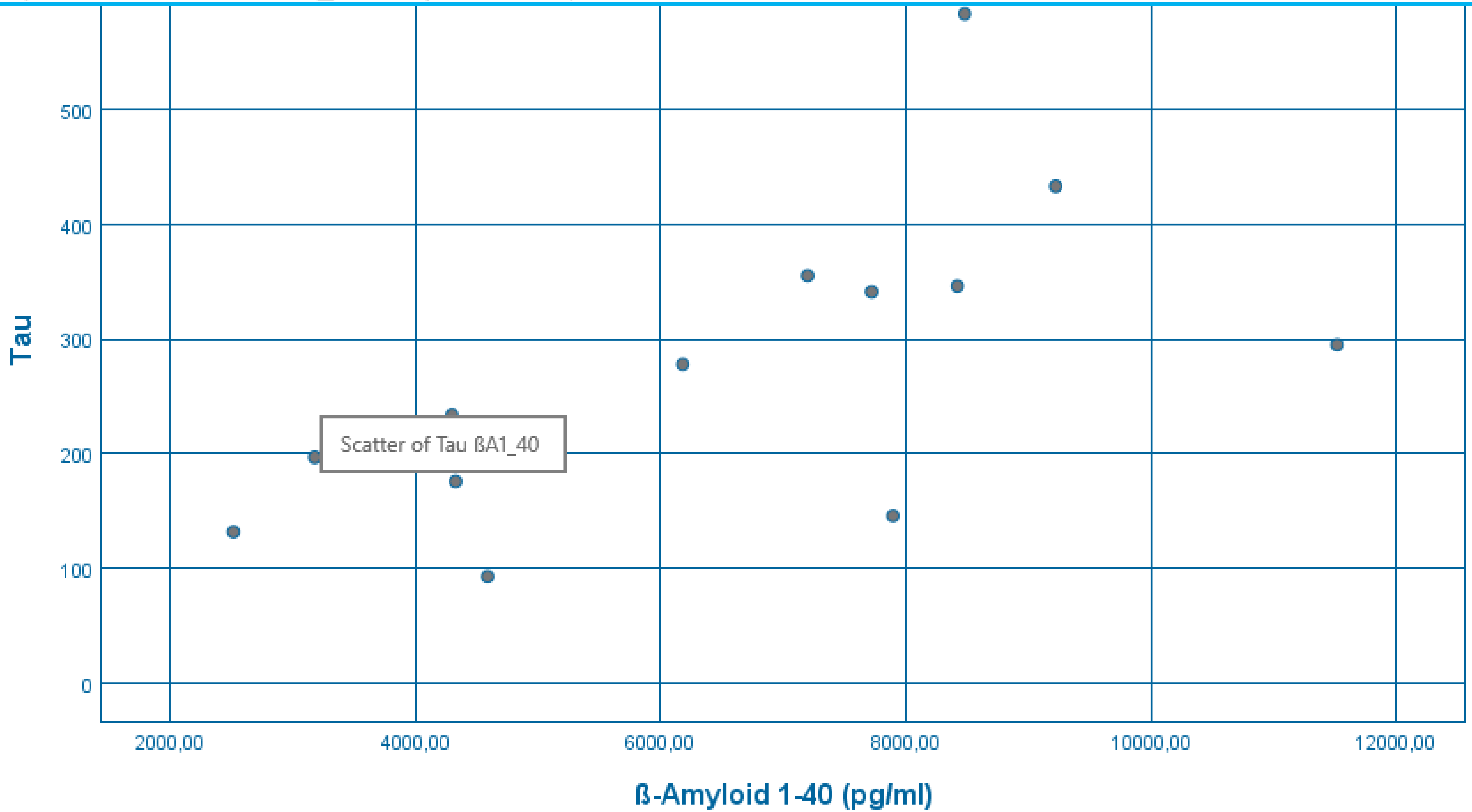

Graph

Graph - Scatter of pTau181 ßA1_40 - September 25, 2019

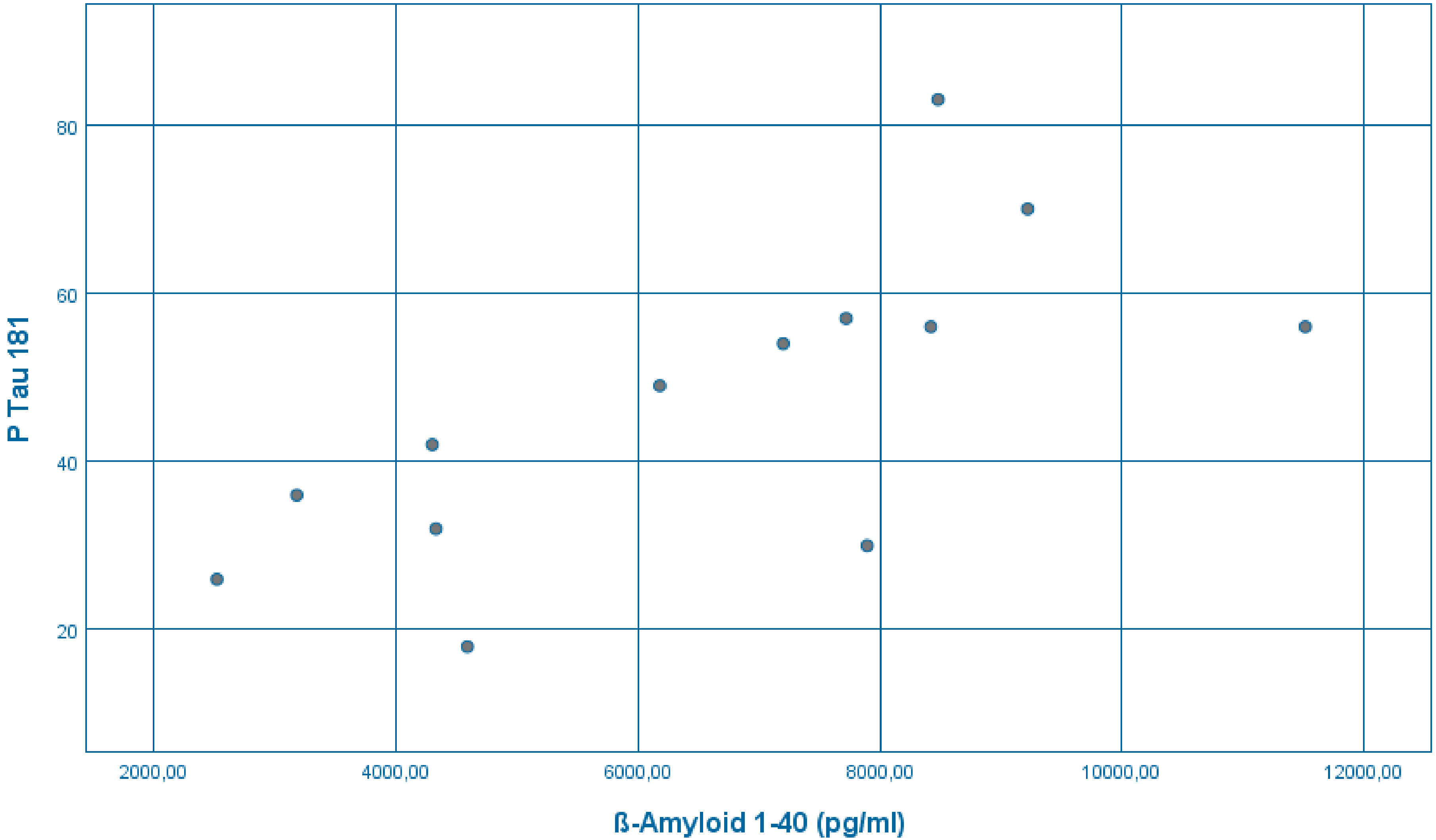

Graph

Graph - Scatter of pTau181 Tau - September 25, 2019

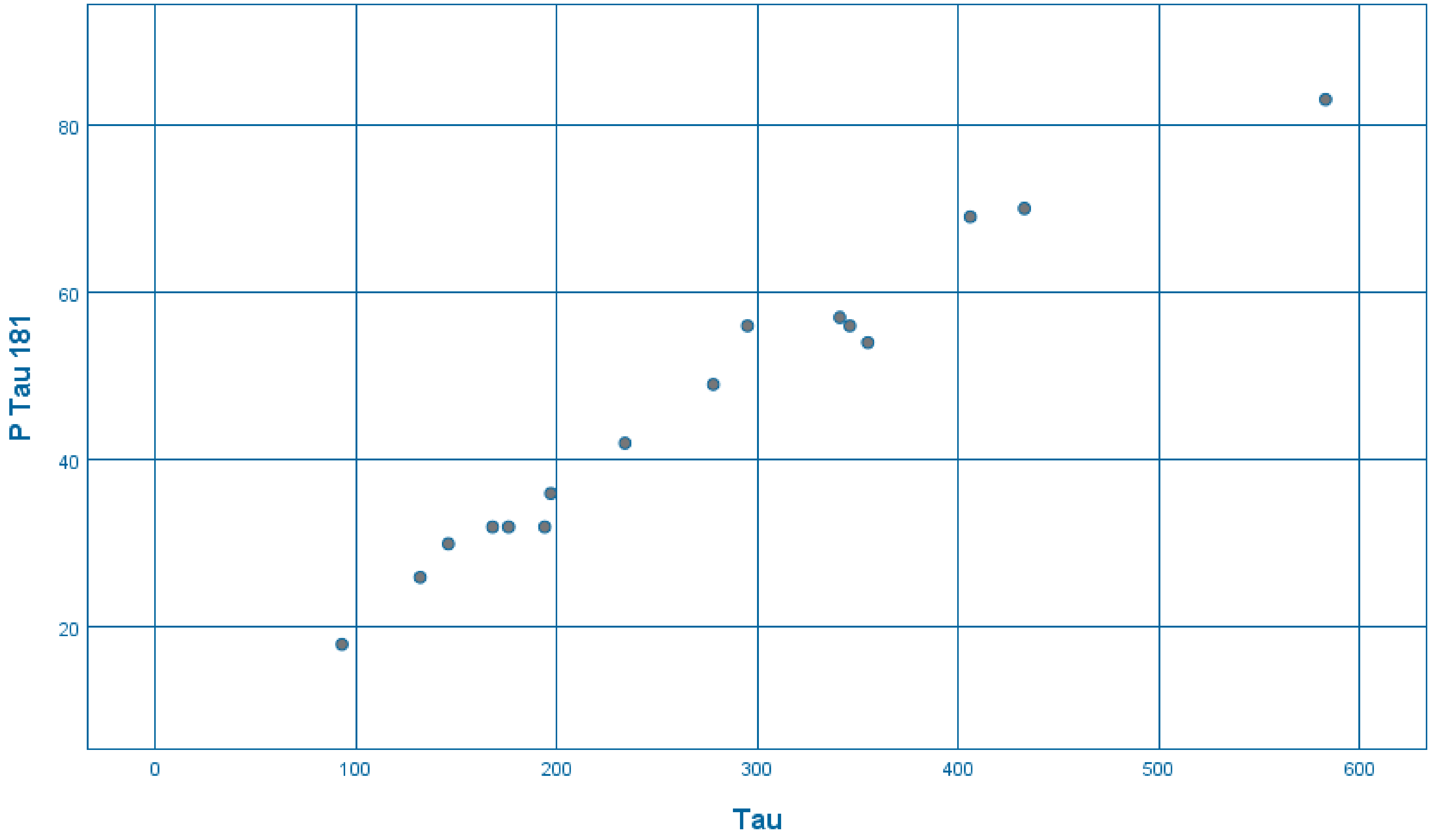

Graph

Graph - Scatter of ßA1_40 ßA1_42 - September 25, 2019

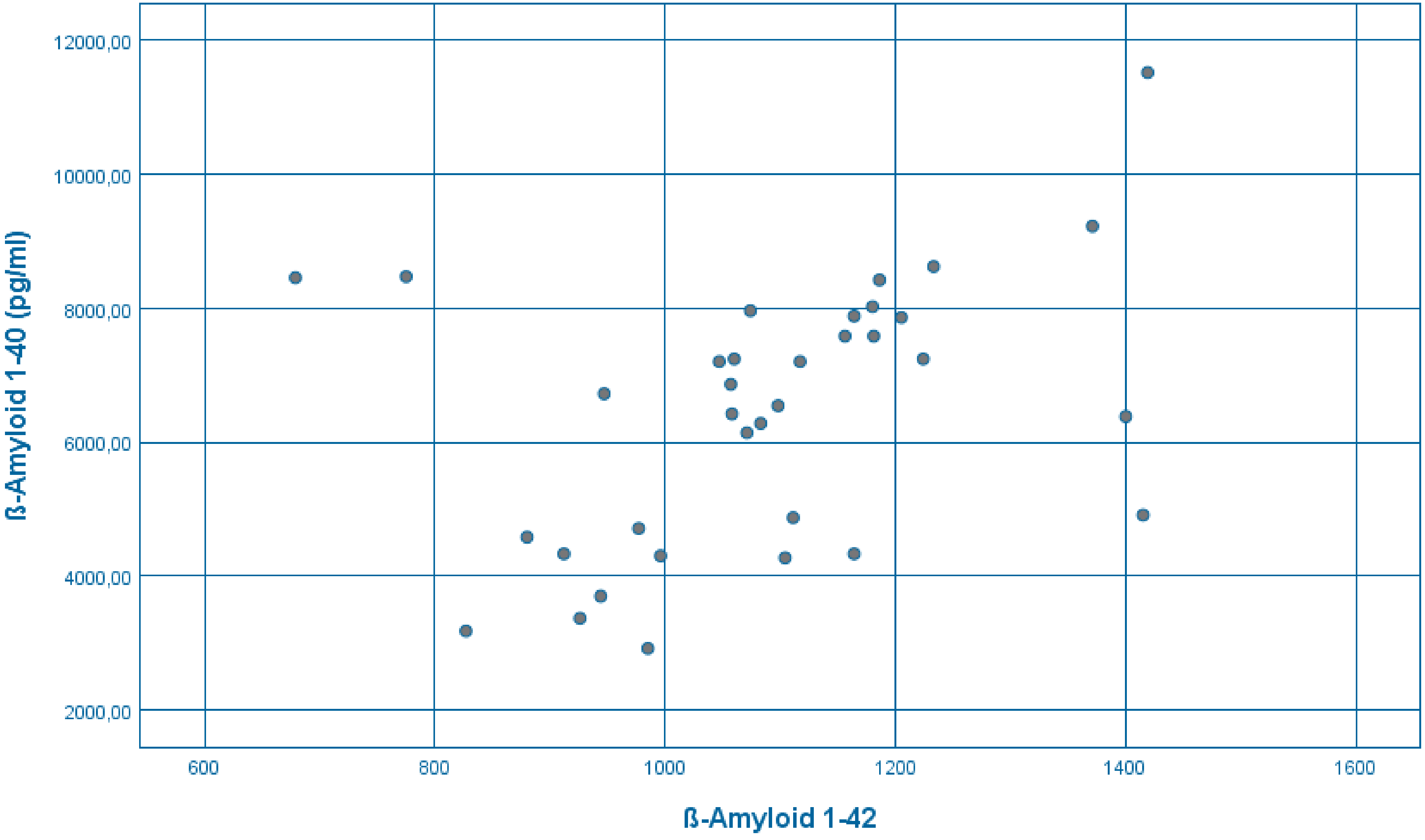

Graph

Graph - Scatter of Tau ßA1_42 - September 25, 2019

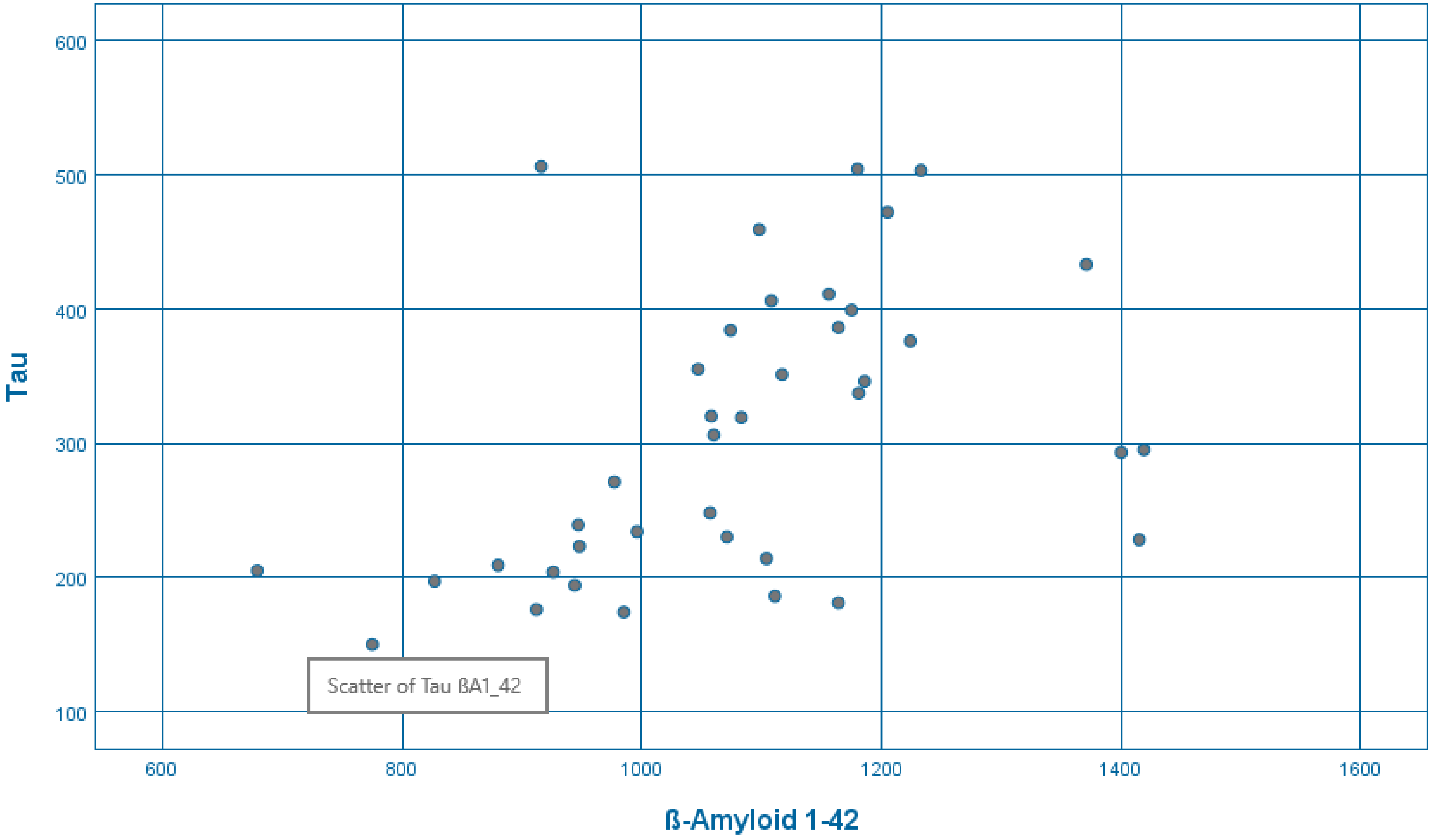

Graph

Graph - Scatter of pTau181 ßA1_42 - September 25, 2019

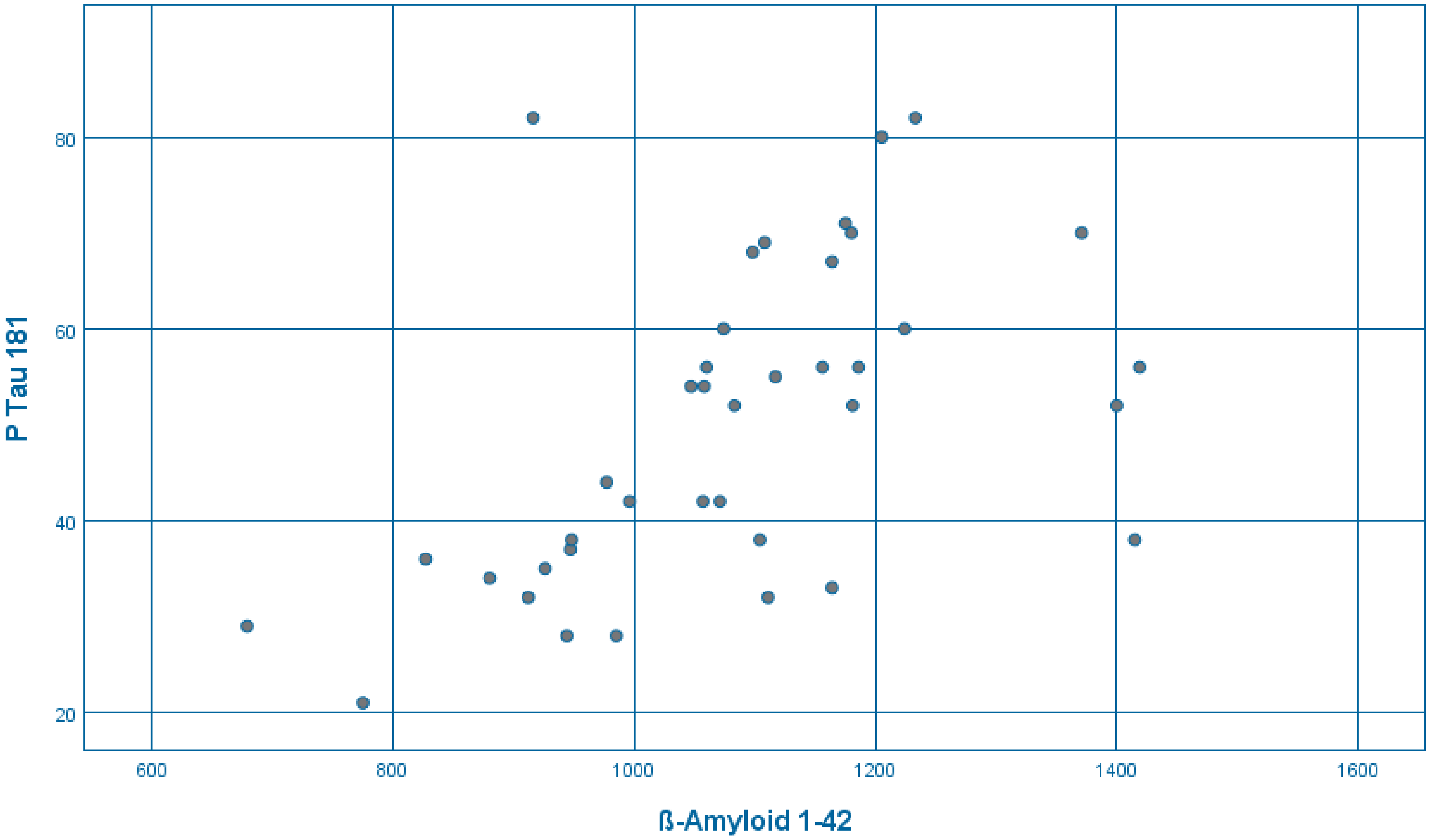

Graph

Graph - Scatter of Tau ßA1_40 - September 25, 2019

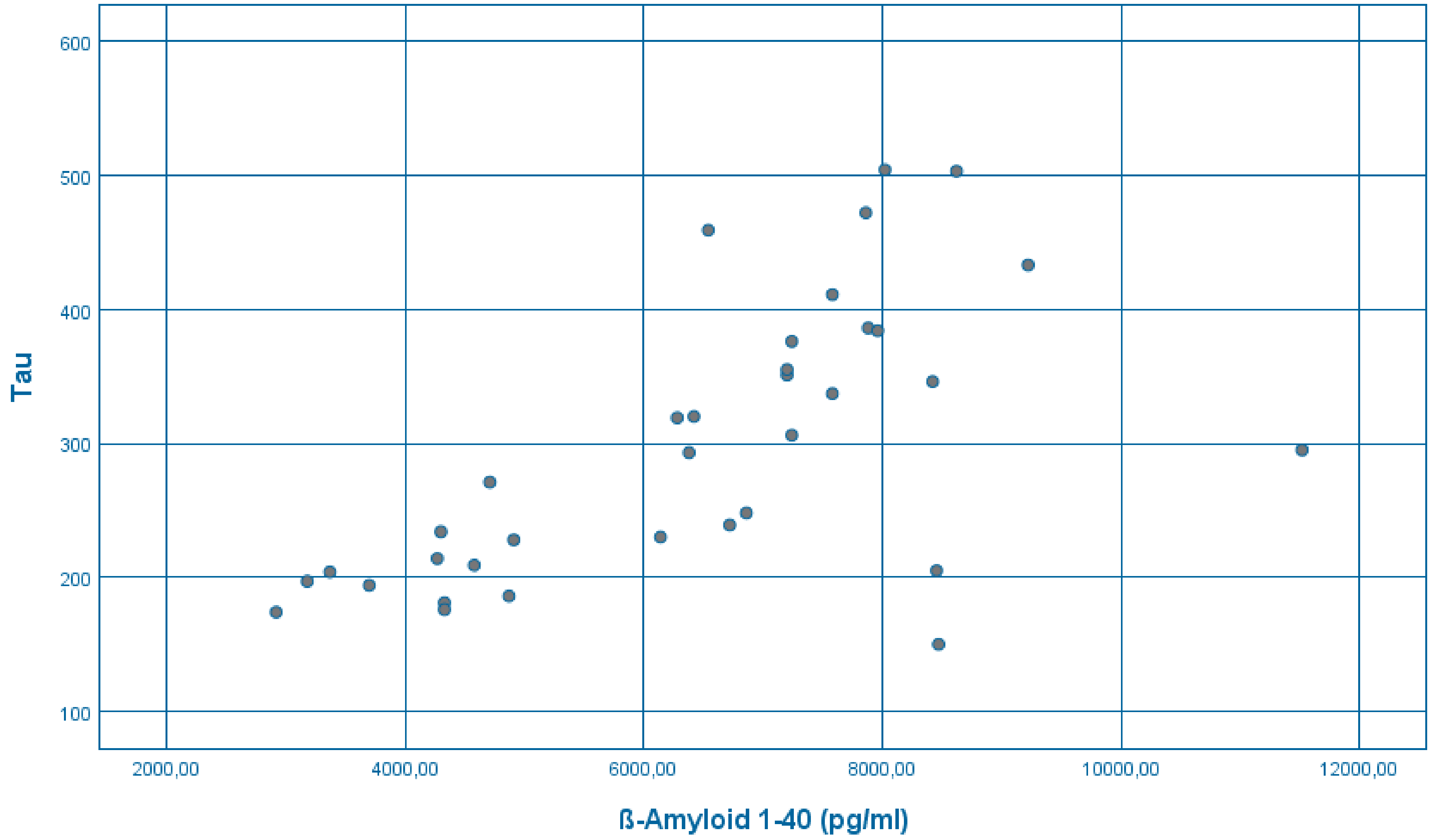

Graph

Graph - Scatter of pTau181 ßA1_40 - September 25, 2019

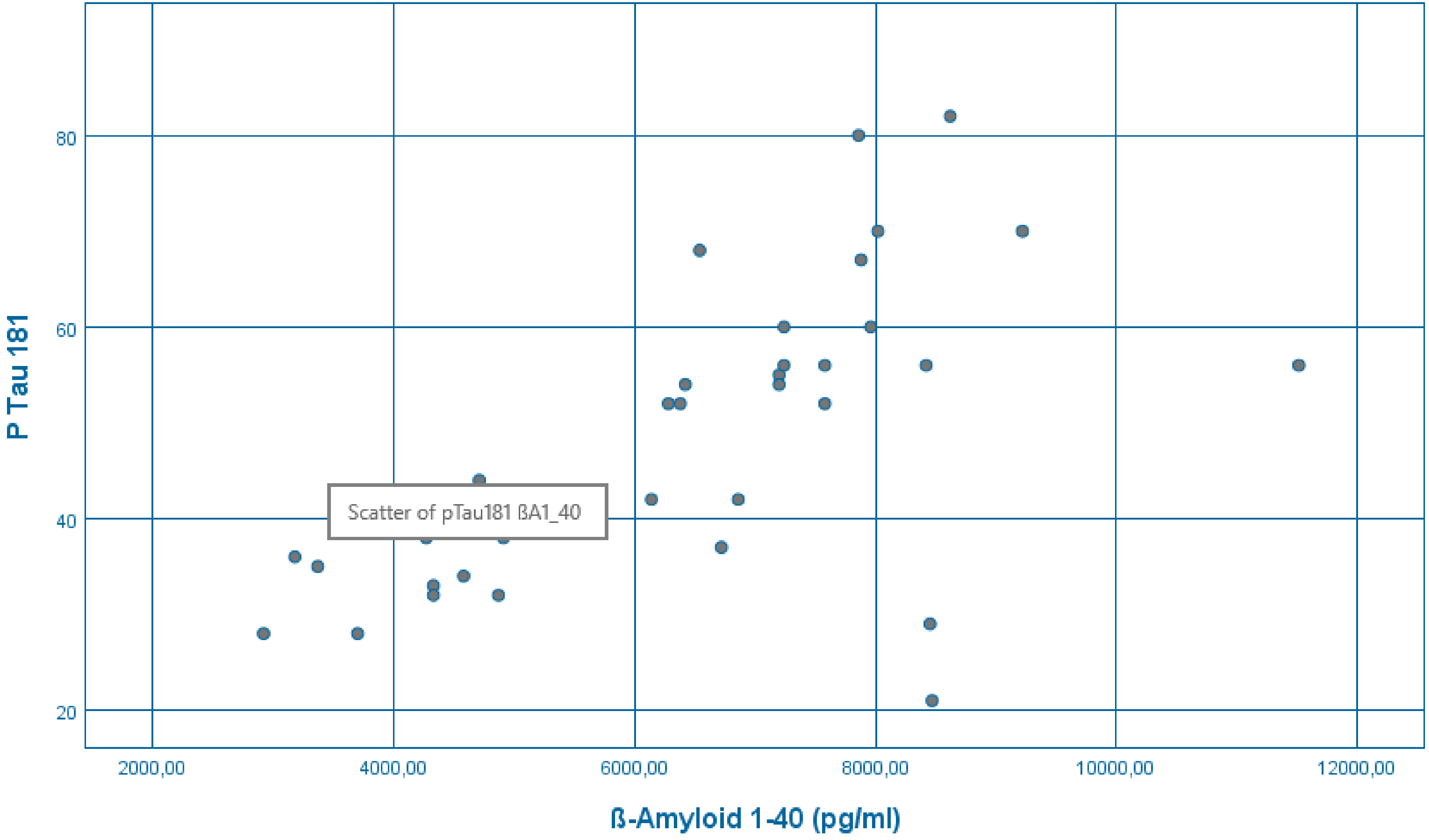

Graph

Graph - Scatter of pTau181 Tau - September 25, 2019

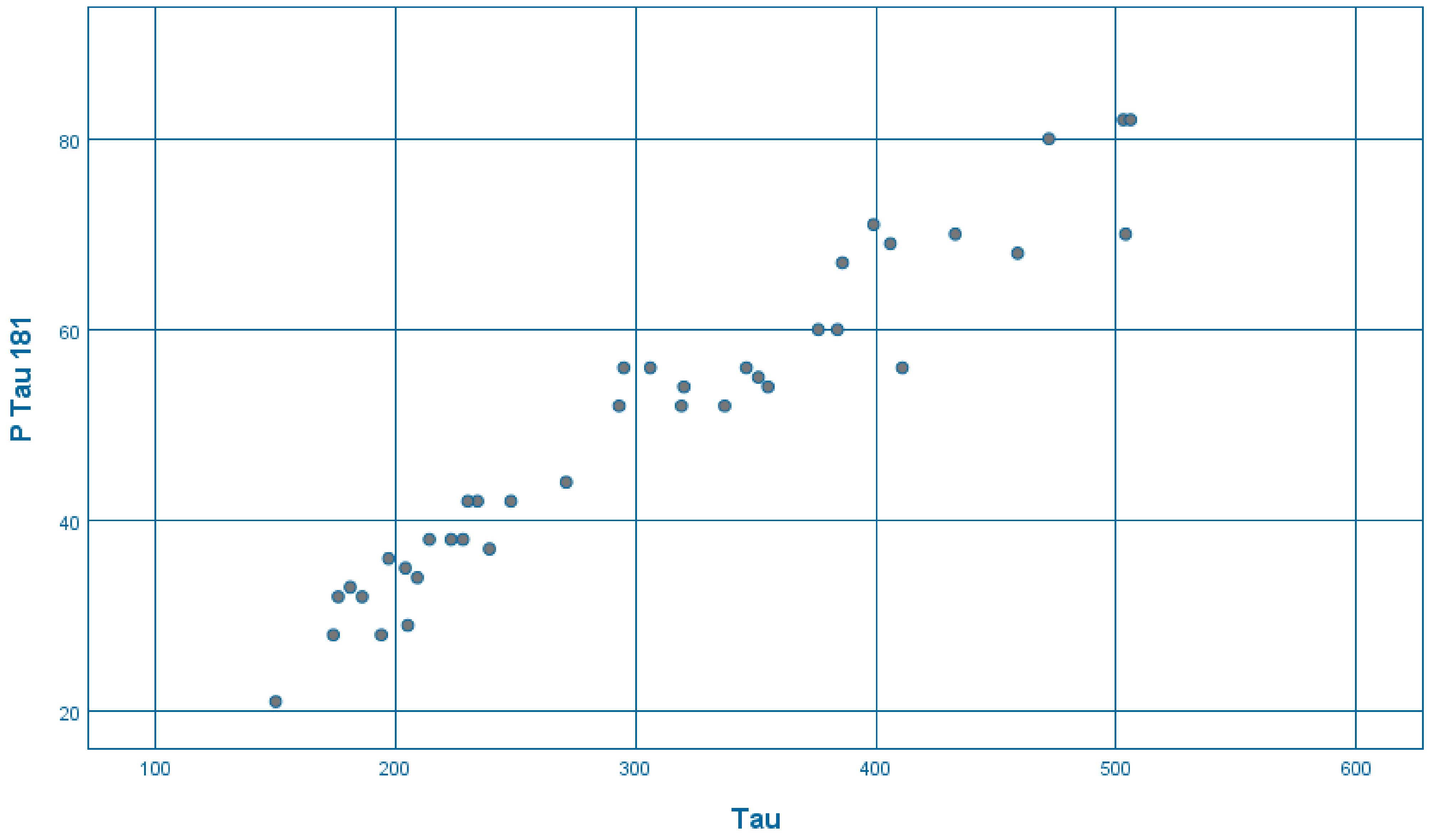

Graph

Graph - Scatter of sFIT_PGF PGF - September 25, 2019

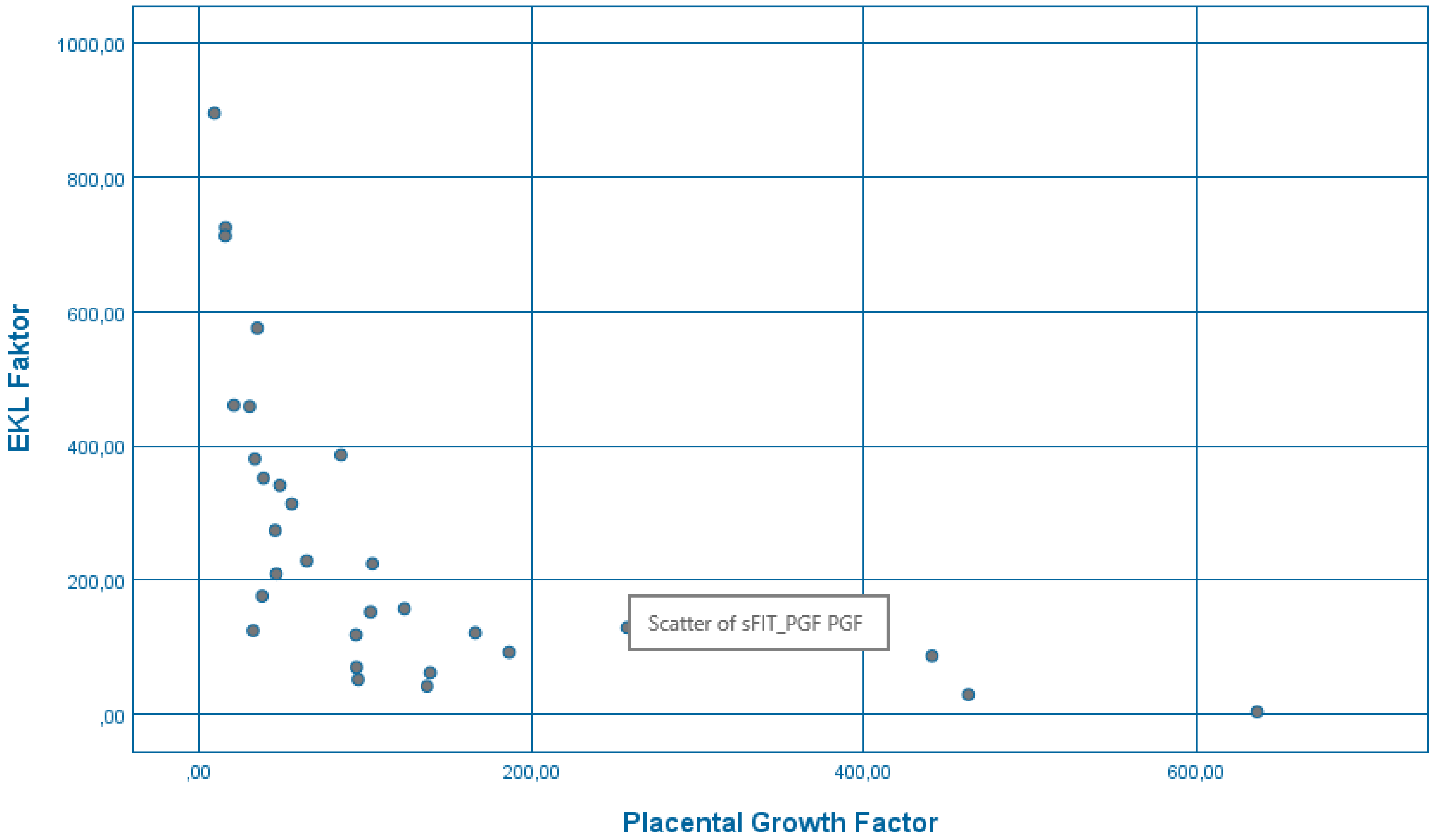

Graph

Graph - Scatter of ßA1_40 sFIT_PGF - September 25, 2019

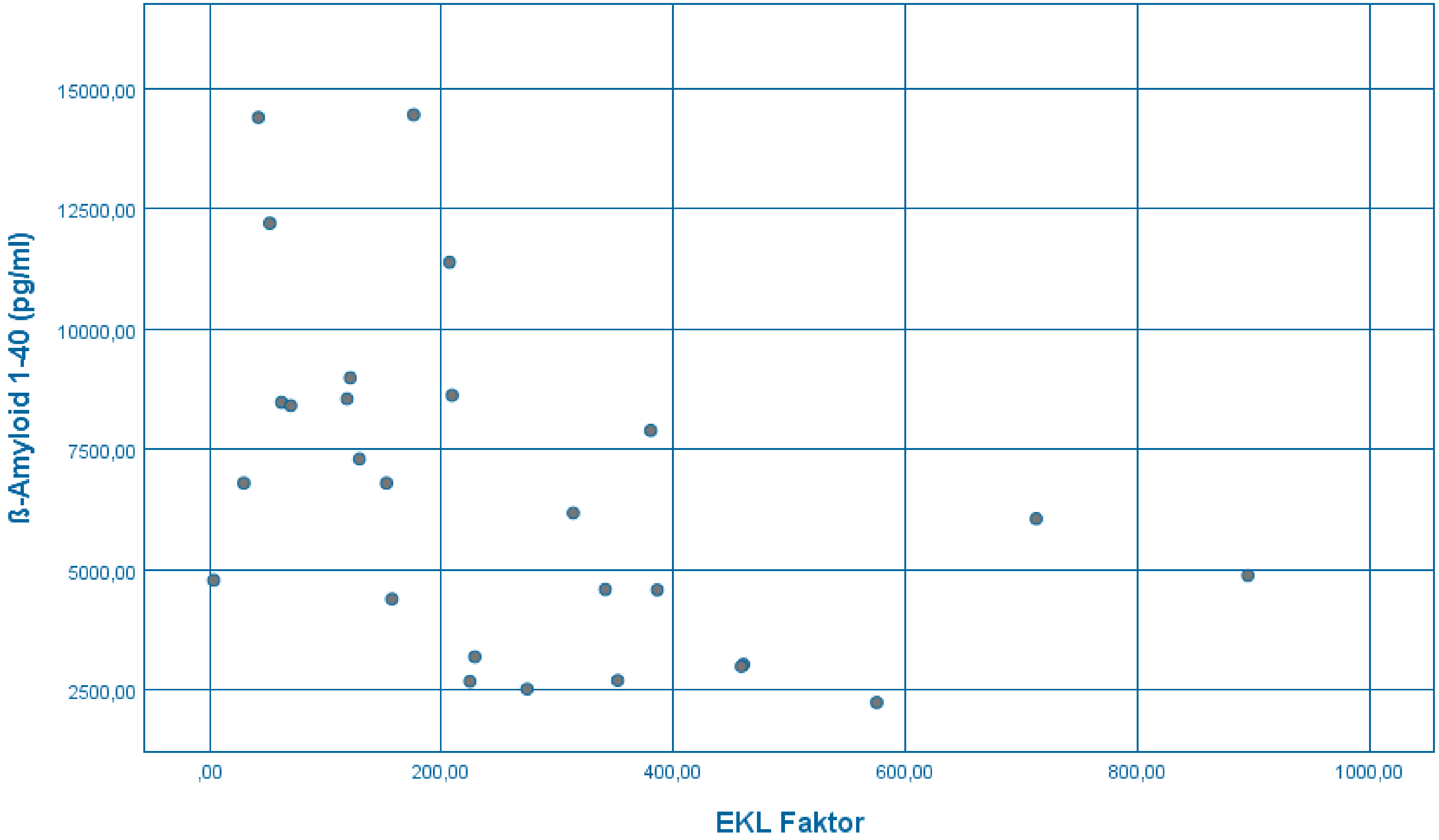

Graph

Graph - Scatter of ßA1_40 ßA1_42 - September 25, 2019

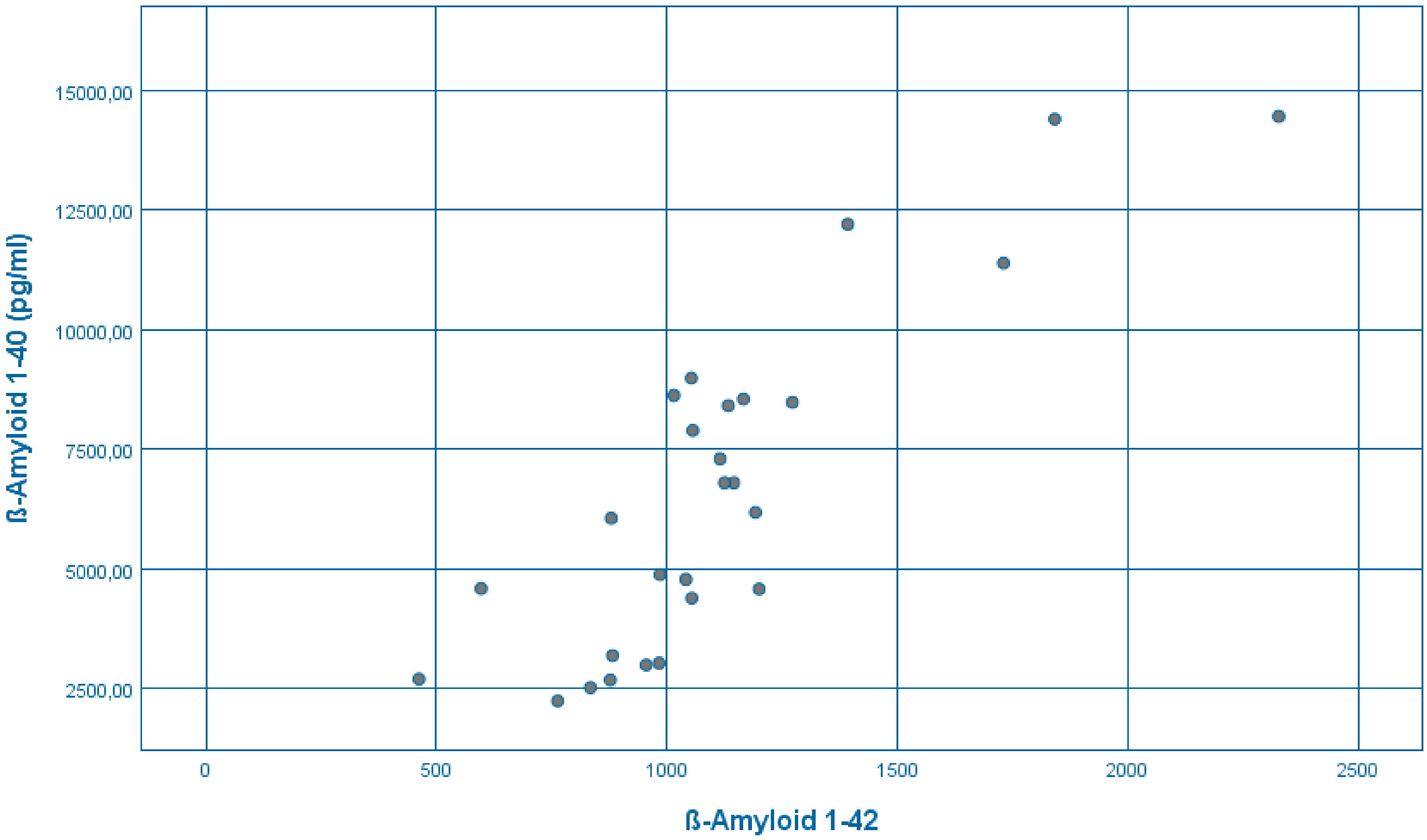

Graph

Graph - Scatter of Tau ßA1_42 - September 25, 2019

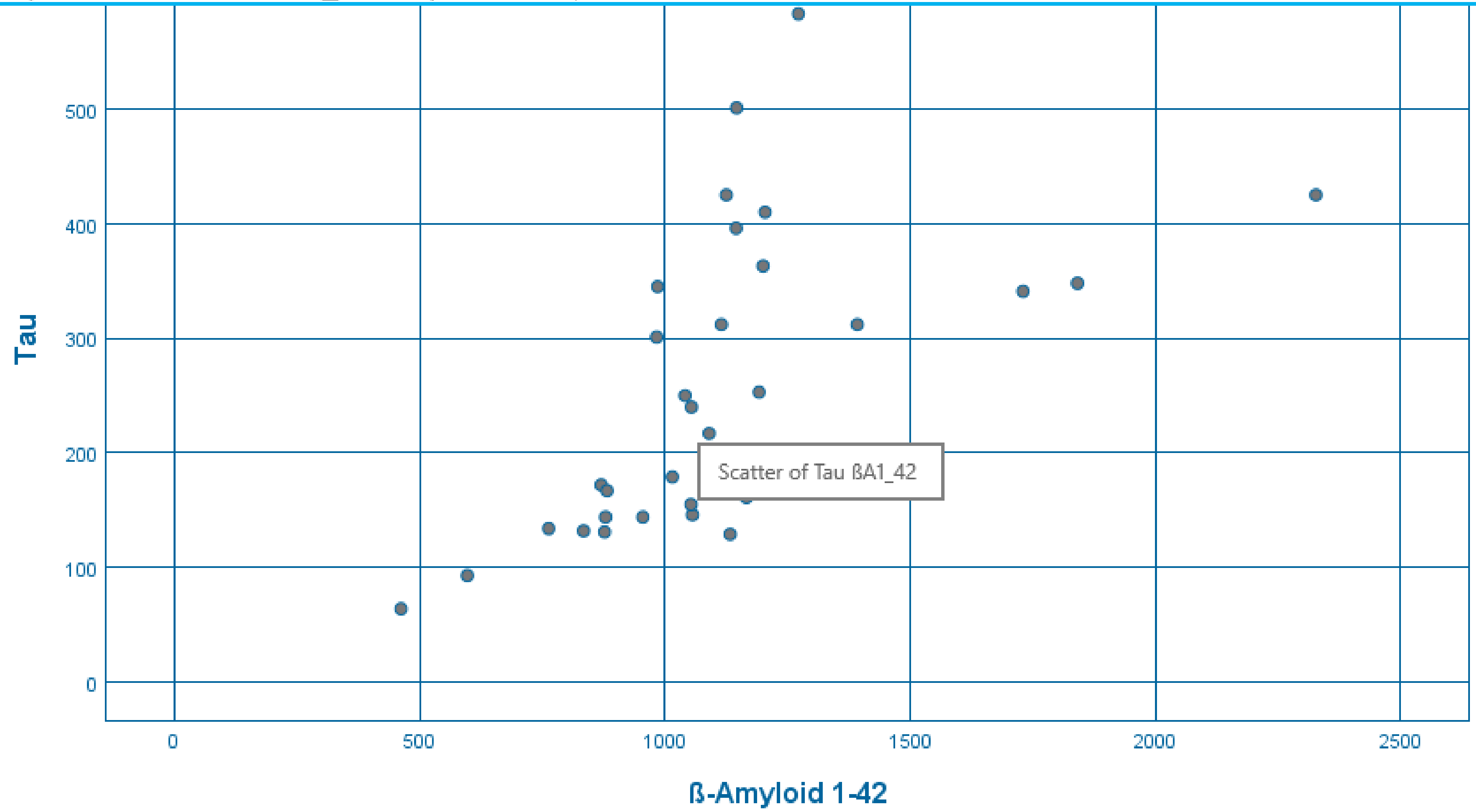

Graph

Graph - Scatter of pTau181 ßA1_42 - September 25, 2019

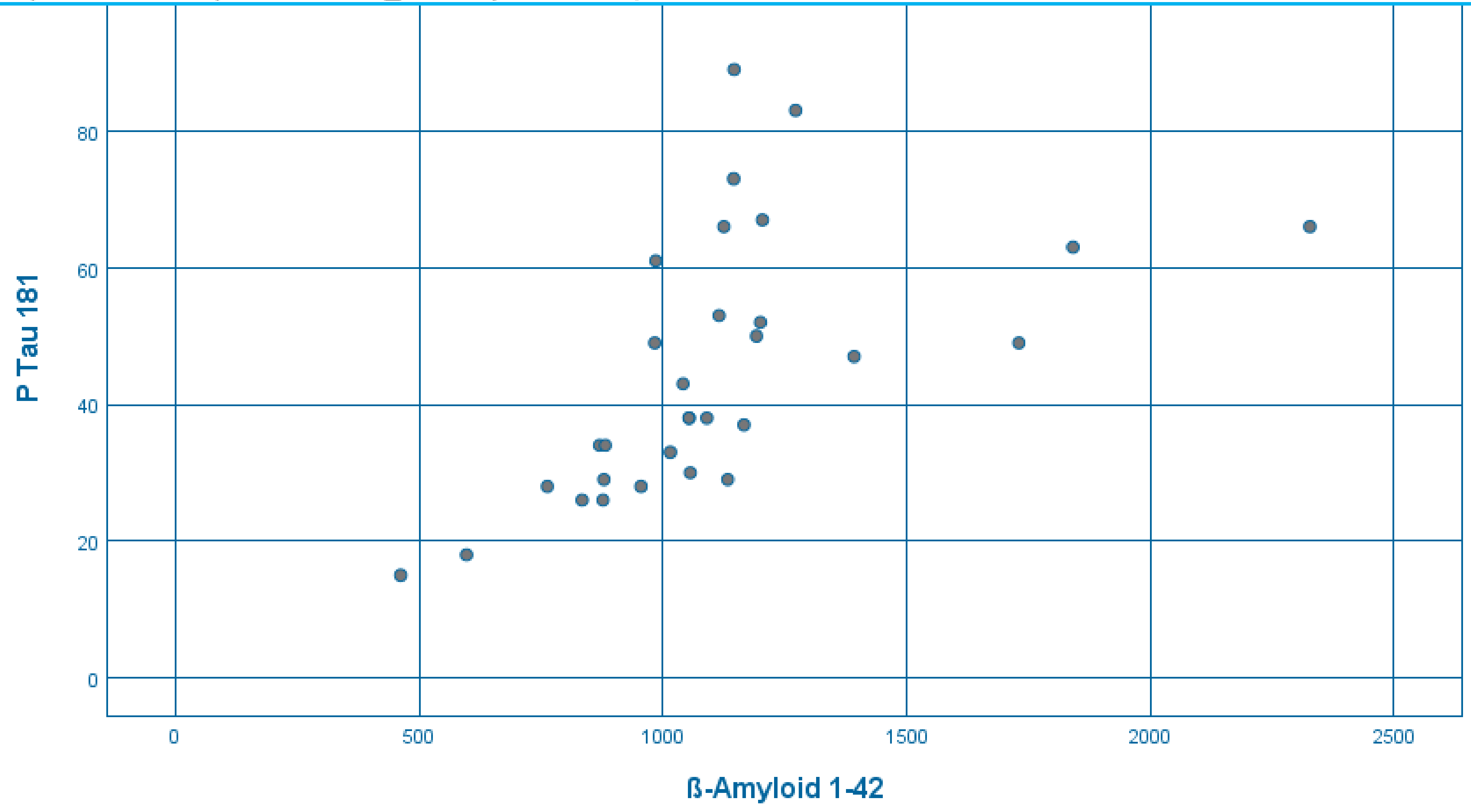

Graph

Graph - Scatter of Tau ßA1_40 - September 25, 2019

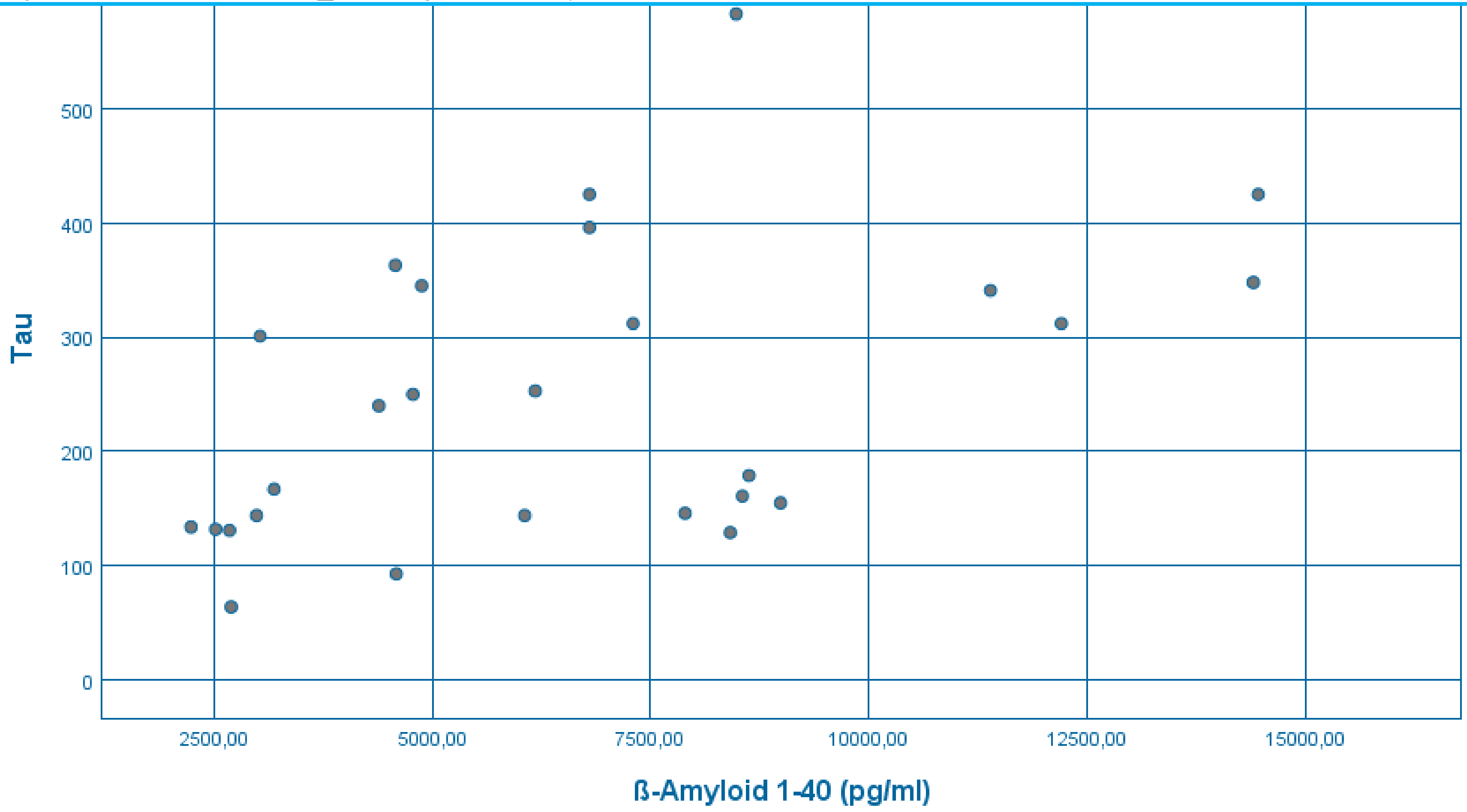

Graph

Graph - Scatter of pTau181 ßA1_40 - September 25, 2019

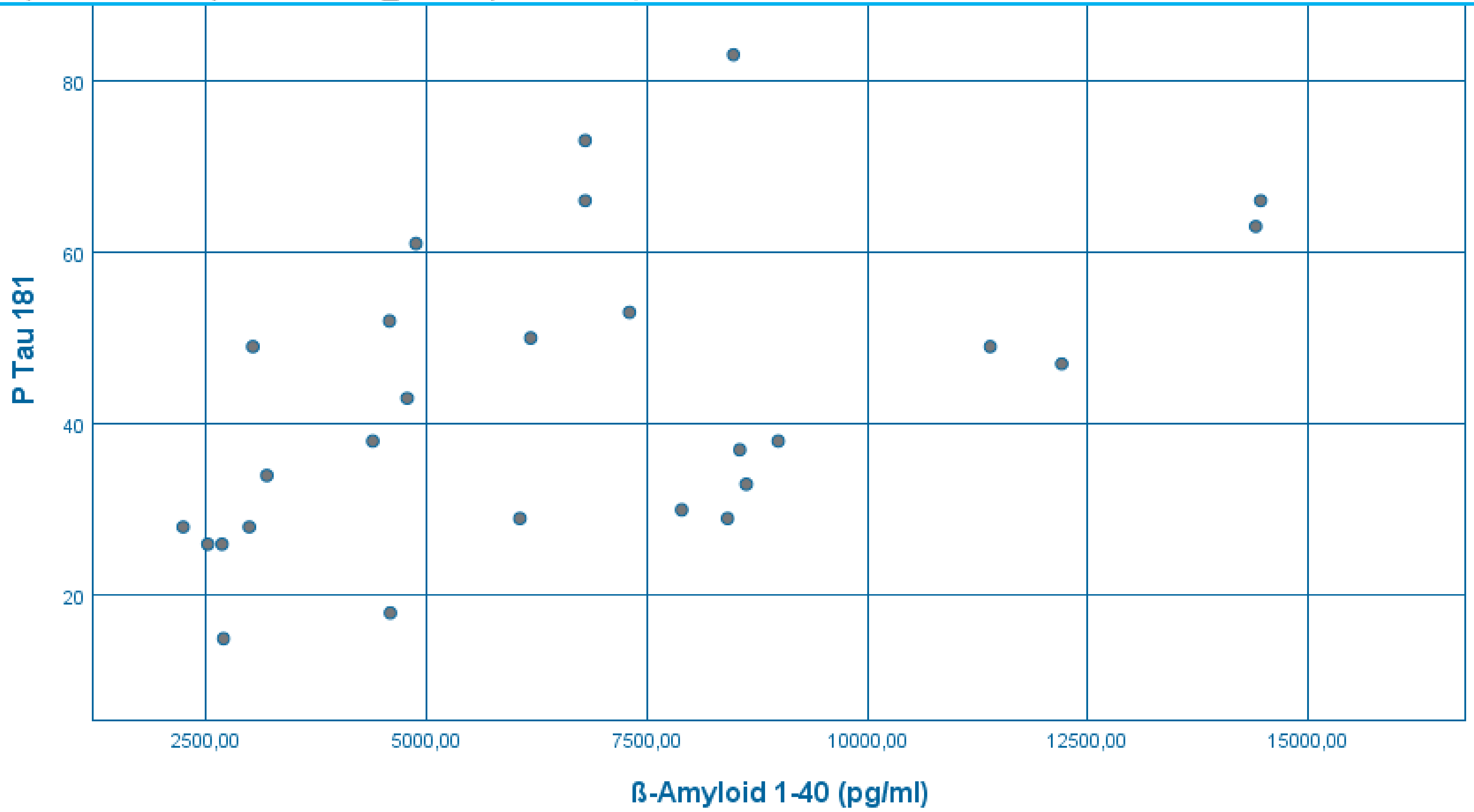

Graph

Graph - Scatter of pTau181 Tau - September 25, 2019

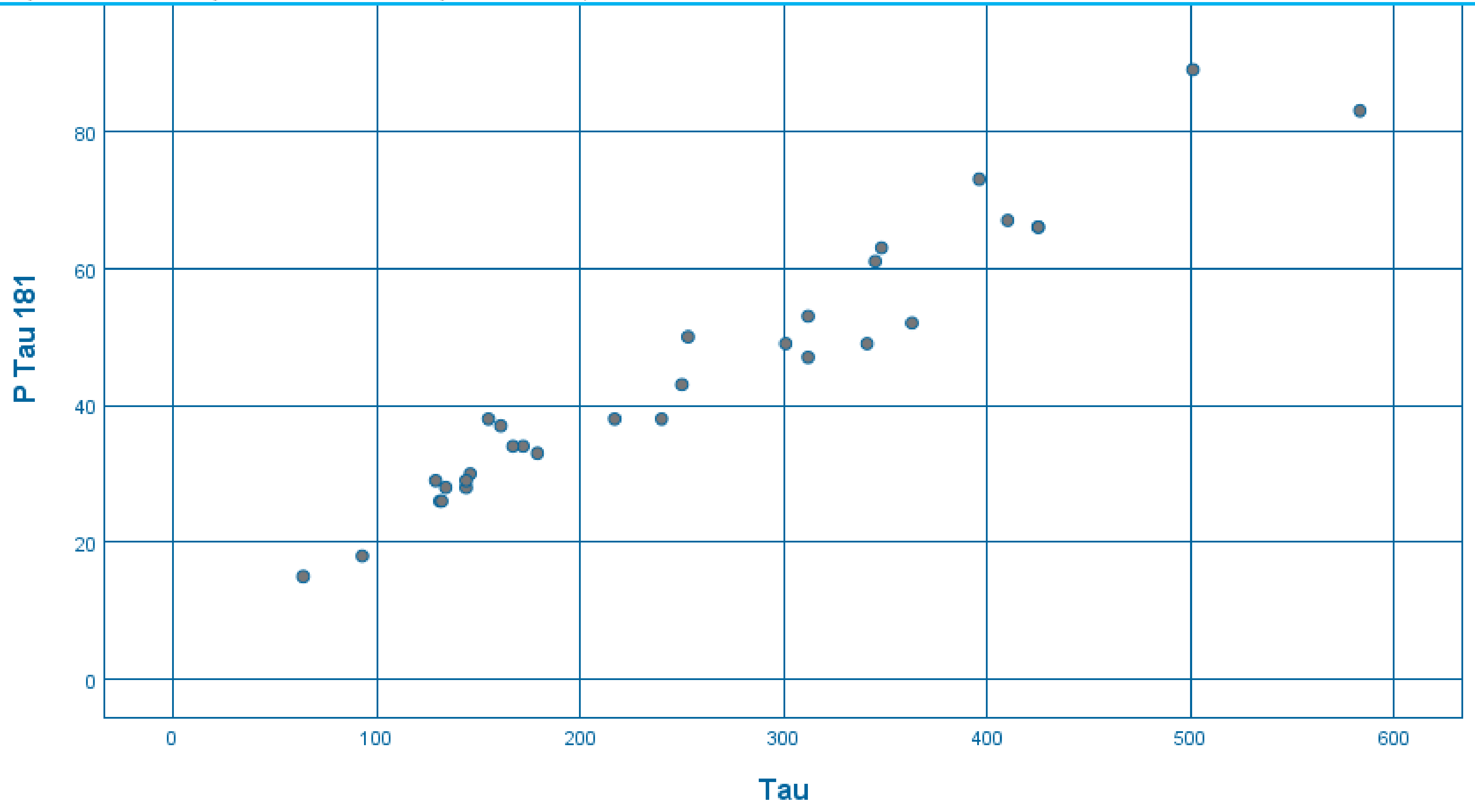

Graph

Graph - Scatter of ßA1_40 Birth_weight - September 25, 2019

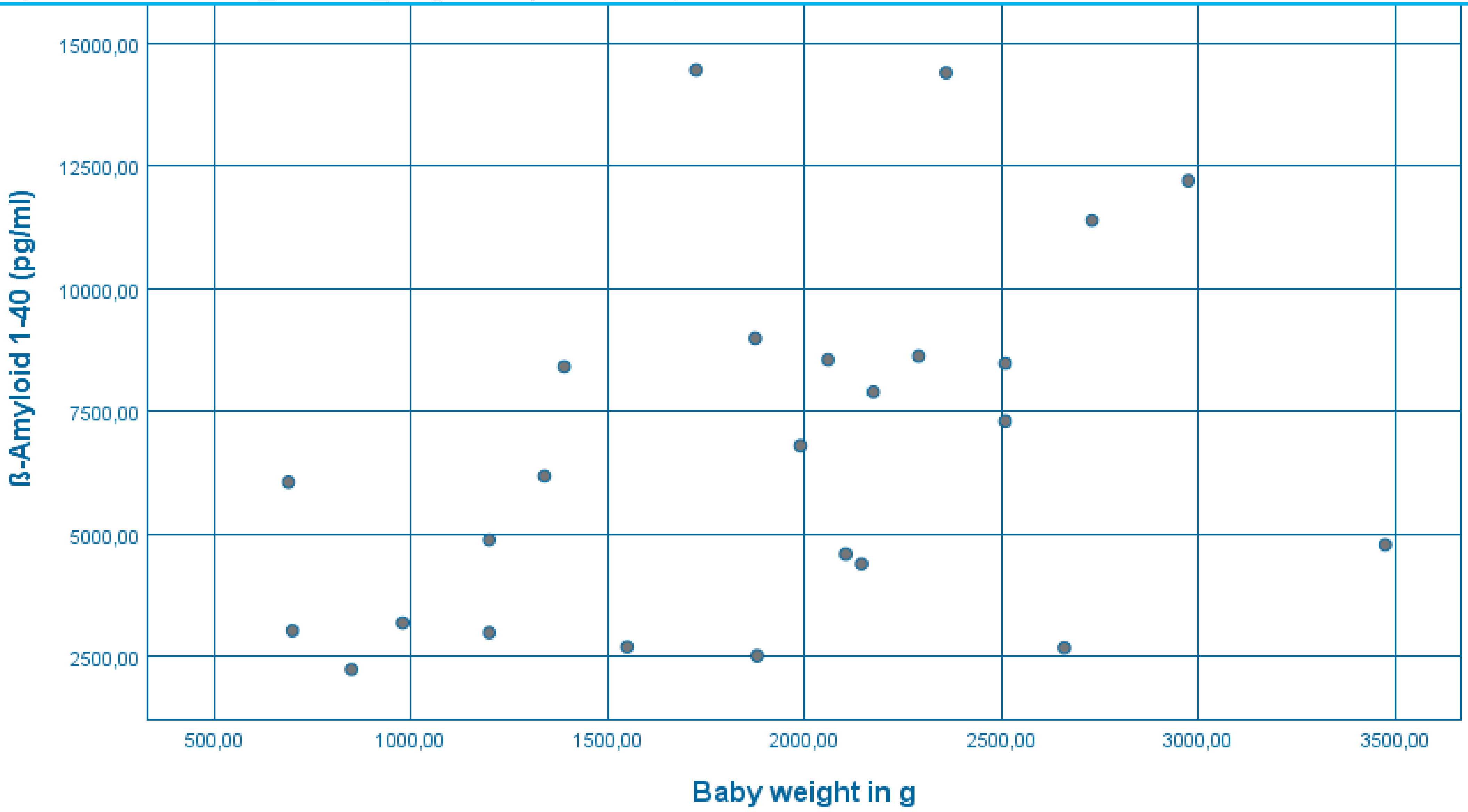

Graph

Graph - Scatter of ßA1_42 pH_child - September 25, 2019

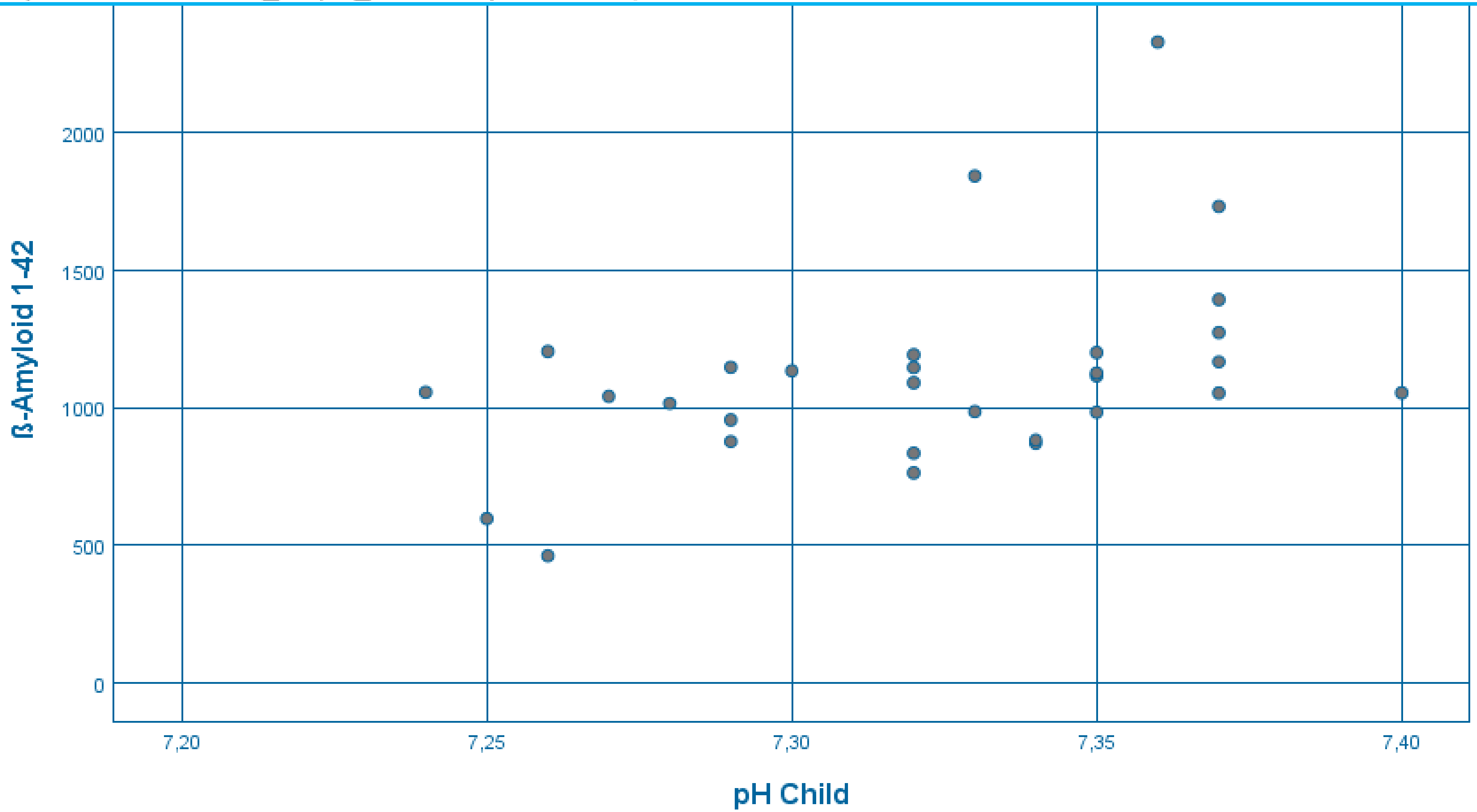

Graph

Graph - Scatter of Tau BE_child - September 25, 2019

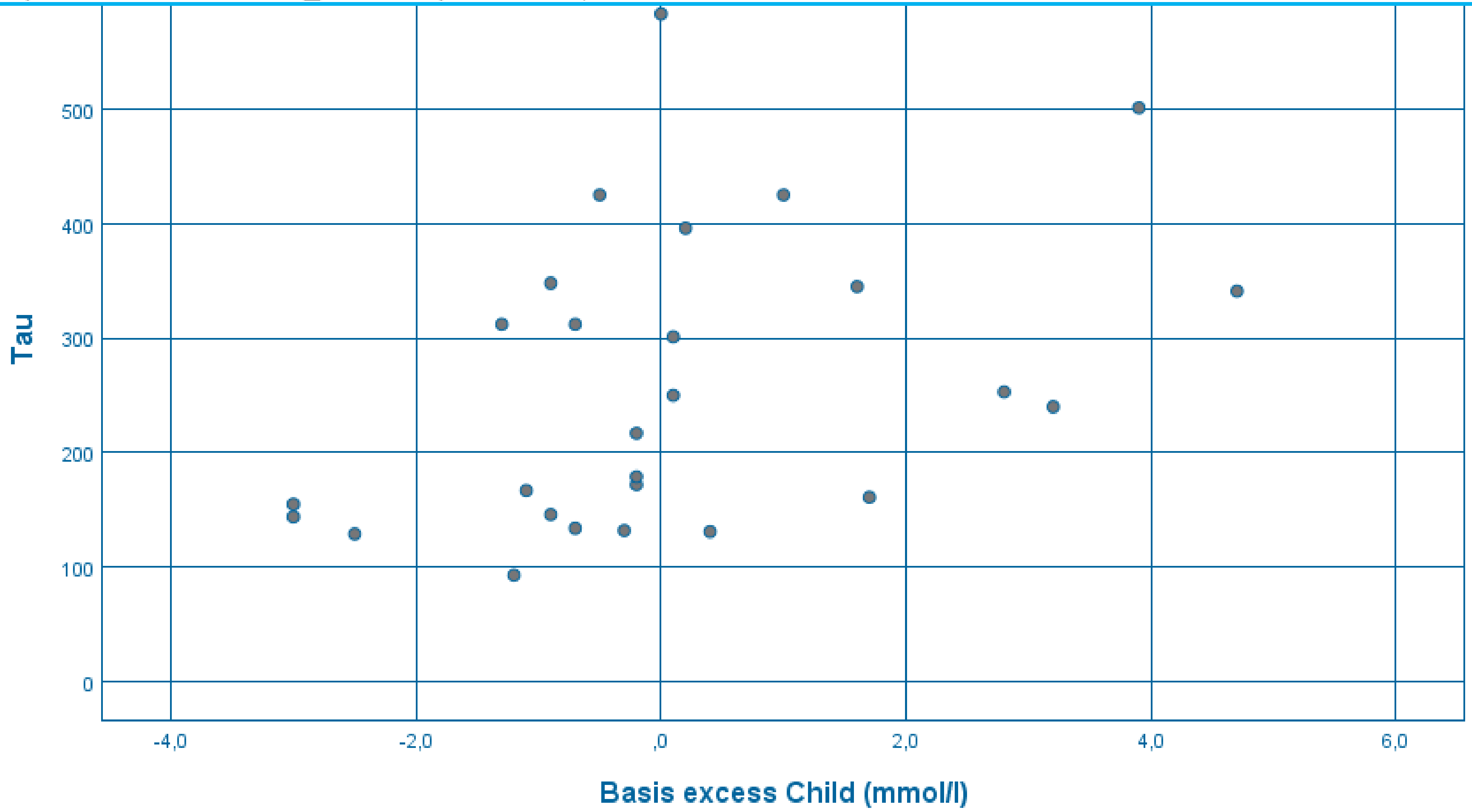

Graph

Graph - Scatter of pTau181 BE_child - September 25, 2019

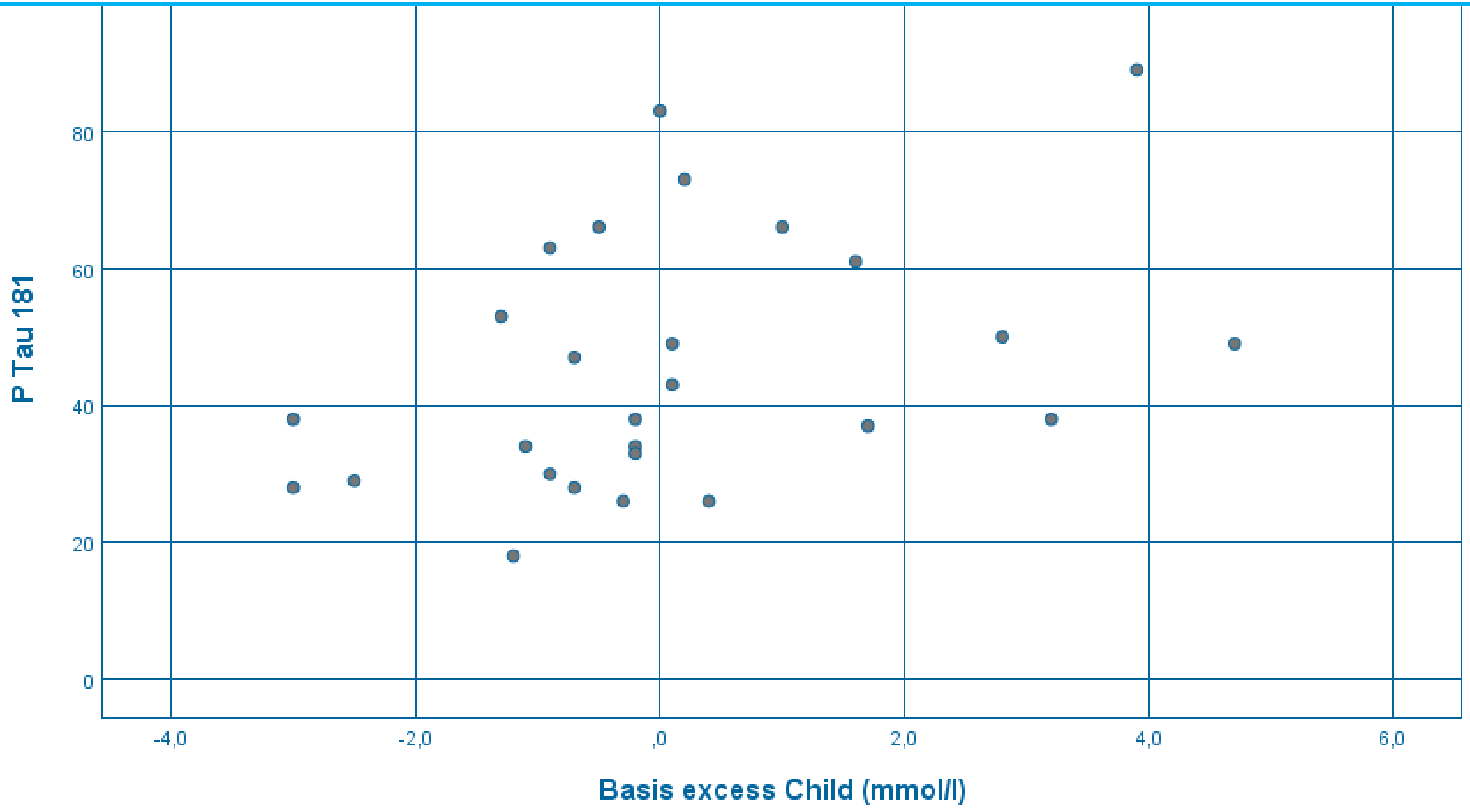

Supplement: Supplementary file 1 — Supplementary Dataset 1. [file 41598_2020_62805_MOESM1_ESM.pdf]
